# Transmission and lesion progression of treponeme-associated hoof disease in captive elk (*Cervus canadensis*)

**DOI:** 10.1371/journal.pone.0289764

**Published:** 2023-08-10

**Authors:** Zachary B. Robinson, Devendra H. Shah, Kyle R. Taylor, Margaret A. Wild

**Affiliations:** 1 Department of Veterinary Microbiology and Pathology, College of Veterinary Medicine, Washington State University, Pullman, Washington, United States of America; 2 School of Veterinary Medicine, Texas Tech University, Amarillo, Texas, United States of America; 3 Washington Animal Disease Diagnostic Laboratory, Washington State University, Pullman, Washington, United States of America; Colorado State University College of Veterinary Medicine and Biomedical Sciences, UNITED STATES

## Abstract

Treponeme-associated hoof disease (TAHD) is a debilitating disease of free-ranging elk (*Cervus canadensis*) in the northwestern U.S. While treponemes are associated with lesions, the etiology and transmissibility between elk are unknown. Our objective was to determine whether the disease can be environmentally transmitted to captive elk. Four individually housed treatment elk and 2 control elk were challenged with soil mixed with inoculum prepared from free-ranging elk hooves from TAHD-positive elk or autoclaved hooves from normal elk, respectively. The inoculum for each group was applied to the interdigital space and added to pre-existing soil in each pen. Eight challenges were conducted at 1–4-week intervals and lesion development was assessed during a 138-day challenge period that was followed by a 170-day monitoring period to document lesion progression. All treatment elk, but no control elk, developed gross and histologic lesions consistent with TAHD. *Treponema* phylotypes similar to those in bovine digital dermatitis in cattle were detected using 16S rRNA gene amplicon sequencing from lesions in all treatment elk, but no control elk, during the challenge period. Lesions progressed from ulcerations in the interdigital space to extensive ulceration and underrunning of the hoof capsule by 35 and 173 days following the initial inoculation, respectively. Lameness in treatment elk was correlated with lesion development (R = 0.702, p≤0.001), and activity of infected elk was reduced during the challenge (p≤0.001) and monitoring periods (p = 0.004). Body condition was significantly lower in treatment than control elk 168 days following the initial inoculation (p = 0.05) and at each individual elk’s study endpoint (p = 0.006). Three of 4 treatment elk were euthanized when they reached humane endpoints, and one elk recovered. These results provide direct evidence that TAHD is a transmissible infectious disease in elk. As such, actions that reduce transmission risk can support disease management and prevention.

## Introduction

Treponeme-associated hoof disease (TAHD) is an emerging disease threatening elk (*Cervus canadensis*), a wildlife species with significant ecological, economic, and cultural value. Clinical signs consistent with TAHD were initially reported in southwestern Washington, USA, in the early 2000s and increased markedly in 2008 [1]. Since then, TAHD has been reported over a broader geographic area of Washington, and portions of Oregon, Idaho, and California [2].

Treponeme-associated hoof disease exhibits characteristic gross and histologic foot lesions. Gross lesions can be graded I through IV that are presumed to reflect progression in severity over time [3]. Grade I-II lesions include ulcerations usually originating in the interdigital space (IDS), with limited extension to the hoof capsule characterizing grade II. Grade III lesions are characterized by extensive ulceration and underrunning of the heel-horn junction, while sloughing of the hoof capsule occurs with grade IV lesions [3]. Histologic lesions are characterized by suppurative inflammation and the presence of invasive spirochetes [3] presumptively identified as treponemes [4]. Histologic lesions show similarities to those described for bovine digital dermatitis (BDD) in cattle [5] and contagious ovine digital dermatitis (CODD) in sheep [6]. Treponemes are considered critical components found with other commonly identified bacteria in BDD [7] and CODD [8]. Although the etiology of TAHD has not been established, treponemes have been a prominent feature in elk hoof lesions [2–4].

Due to the presence of *Treponema* phylotypes similar to those associated with BDD [4] and geographic expansion of observed cases, TAHD is hypothesized to be an infectious, transmissible disease. However, competing hypotheses argue that bacterial infection is coincident or secondary to other inciting factors, such as exposure to herbicides or nutritional deficiencies [9]. Bovine digital dermatitis is transmitted directly from infected to uninfected cattle and indirectly by contaminated fomites and environmental slurry [10–12]. Previous attempts to reproduce TAHD in a domestic sheep model included experimental application of lesion material from TAHD-positive elk hooves to scarified skin on the ventral pastern of domestic sheep [13]. This experimental model resulted in development of gross lesions that were inconsistent with TAHD, however, presence of treponemes and histologic lesions similar to TAHD were observed [13]. It is currently unknown if TAHD can be experimentally reproduced in elk. Our objective was to determine whether TAHD is environmentally transmissible to elk in a captive setting. We hypothesized that following experimental exposure of captive elk to TAHD-positive hoof material mixed with soil, elk would develop gross and histologic lesions indistinguishable from those in free-ranging elk with TAHD and that treponemes would be consistently present in these lesions. Further, we anticipated that hoof lesions would progress in severity from grade I to IV over time, leading to increased lameness and decreased body condition.

## Material and methods

### Experimental animals and housing

Experimental animal use was approved in writing (Animal Subject Approval Form number 6707) by the Washington State University Institutional Animal Care and Use Committee. Sample collection was conducted while elk were under sedation. Analgesics were administered to reduce pain and all efforts were made to minimize suffering. Six (5 male, 1 female), 1- to 2-year-old Rocky Mountain elk (*C*. *c*. *nelsoni*) obtained from Kittitas County, WA, an area where TAHD is not considered endemic, were used in the study. Elk were individually housed in outdoor concrete-floored pens (4.9m x 6.1m) with wood shaving bedding at the Elk Research Facility, Washington State University, Pullman, WA. A maintenance diet of orchard grass supplemented with pelleted feed (Mazuri Exotic Animal Nutrition, St. Louis, Missouri, USA) was provided along with ad libitum access to mineralized salt blocks (American Stockman, Compass Minerals America, Overland Park, Kansas, USA) and water.

Four to 5 days prior to study initiation, experimental housing conditions were modified through addition of soil to each pen. Soil was added at a depth of 5 cm to cover about one third (2.7 m^2^) of each pen and retained for the duration of the study. An irrigation system at the rear of each pen was used to keep soil moistened during the study. Soil source and maintenance of moisture were intended to simulate the natural environmental conditions in southwestern Washington where TAHD is endemic. In addition to serving as a source of soil for the pens, soil from the same source was also used to mix with inoculum to prepare experimental challenge material (additional details below). Soil was obtained from a natural area where elk, but not livestock, range and TAHD is endemic near Mount St. Helens in southwestern Washington (46.3407659, -122.5248923). Soil was collected from the surface to a depth of about 30 cm over a 25-m^2^ area and mixed using heavy machinery. An aliquot of soil (500 mL) was collected and analyzed for presence of pesticides and herbicides by Anatek Labs Incorporated (Moscow, Idaho, USA). The chemical screen included aminomethylphosphonic acid, glyphosate, clopyralid, triclopyr, 2,4-D, atrazine, hexazinone, imazapyr, metsulfuron-methyl, and sulfometuron-methyl. Soil underwent a ≥15-day solarization process at 27 to 37°C to reduce potential pathogen abundance [14] prior to use. Solarization decreased the risk of exposure of study elk to pathogens other than those from experimental challenges.

### Experimental design

Elk were randomly assigned to treatment groups and housed individually in pens from October 2020 to September 2021. A treatment group (n = 4) was exposed to an inoculum containing macerated TAHD-positive elk hoof tissue mixed with soil while a control group (n = 2) was exposed to autoclaved and macerated grossly normal elk hoof tissue mixed with soil. The soil was included with hoof tissue in a challenge inoculum to simulate the transmission of TAHD under natural environmental conditions. On each challenge day, frozen hooves from harvested free-ranging elk were thawed, sampled, and processed to prepare inoculum (additional details below). Each control and treatment elk was examined and sampled prior to exposure to this inoculum. When persistent gross lesions consistent with TAHD were observed in all treatment elk, we ceased challenges and commenced a monitoring period. During the challenge and monitoring periods, elk were observed for behavioral changes. Animal behavior was evaluated for attitude, activity, and respiration for at least 2 minutes daily and appetite was evaluated based on 24-hour feed consumption. Attitude was categorized as: bright, alert, responsive (BAR); aggressive; depressed; or moribund. Activity was categorized based on the predominant action observed: calm (standing, standing and slow pacing, walking, or bedded); excited (running or jumping); fast pacing; or reduced (reluctant to rise from bedded or to walk). Elk were designated inactive if bedded, or active if they displayed other activities. Respiration, based on flank movement counted for 1 minute, was categorized as normal (16–24 bpm), tachypnea (>24 bpm), or bradypnea (<16 bpm). Appetite, evaluated based on estimated observed feed consumption, was categorized as normal (all daily feed allocation consumed) or reduced (not all daily feed allocation consumed).

Each elk’s locomotion was assessed for at least 2 minutes daily. Locomotion was scored on a scale of 0–3, where higher scores represented increasing levels of lameness [adapted from 15] (Table 1). The study concluded when 3/4 (75%) treatment elk reached humane endpoints identified in the animal care protocol.

**Table 1:** Locomotion scores.

Category	Definition
0	Sound. Walks confidently, with even weight on all 4 feet; tracks up (hind feet in prints of fore feet); no swinging of legs inward or outward.
1	Imperfect locomotion. May walk cautiously, possibly because of tenderness, OR does not track up, OR legs swing out or in, but no obvious limp.
2	Lame. Definite limp (foot fall uneven, reluctance to bear full weight on affected foot [i.e., toe touching], dew claws on an affected limb do not drop as far) OR arched spine. A favored limb will move more quickly than a lame limb. Speed of the walk not noticeably affected. The animal may hold foot in a toe-touch position at rest but continues to be partially weight-bearing at the walk.
3	Severely lame. Clear difficulty in locomotion and getting up from bedding. Cannot walk at a brisk pace using affected limb. Animal shows obvious signs of limb pain (e.g., avoids bearing weight on the affected limb, 3-legged lame).

Scoring system adapted from [15] used to assess locomotion in captive study elk.

### Inoculum preparation

Treatment inoculum for experimental challenges was prepared using abnormal hooves from 16 free-ranging elk that were harvested or collected by biologists for management purposes in 2020–2021 from areas of Washington where TAHD is endemic [2]. From each free-ranging elk, or case, all 4 feet were collected, except for one case in which only 2 feet were available. Feet were collected proximal to the dew claws and stored at -80°C within 24 hours of the estimated time of death. Control elk hooves were similarly collected from elk with grossly normal hooves.

Treatment inoculum was prepared on each of 8 challenge days using hooves from 2 free-ranging elk (i.e., 2 cases) pooled to create an inoculum. Hooves were thawed by submerging them in water baths maintained at 37°C for 2–3 hours [16]. Each hoof was rinsed with tap water followed by clipping hair in the IDS for visual inspection of lesions. Lesions were photographed, described, and graded (scale of 0-IV; [3]) by a board-certified pathologist. To confirm TAHD status for each case, an 8-mm punch biopsy was collected from a lesion, generally in the IDS, of 1–2 feet and placed in 10% neutral buffered formalin for histologic evaluation. A 5-mm punch biopsy was collected from every foot for 16S rRNA gene amplicon sequencing (hereafter, 16S amplicon sequencing) for detection and quantification of treponemes. These biopsies were collected from a lesion, generally in the IDS, and adjacent to 8-mm biopsy collection sites. Samples were placed in AllProtect tissue reagent (QIAGEN, Germantown, Maryland, USA) and frozen at -80°C until further processing. Remaining foot tissue was used to prepare 2 types of treatment inocula: (i) inoculum for direct application to the IDS of study elk (i.e., direct inoculum) and (ii) inoculum for application to the pen soil (i.e., pen inoculum). The 2 types of inocula were intended to optimize the likelihood of exposure and potential for transmission: direct inoculum ensured that contact with the foot occurred, while application of pen inoculum supplemented the opportunity for additional contact as elk walked in the pen. Control inoculum, for both direct and pen inocula, was prepared similarly except the inoculum was autoclaved and frozen until thawed the night before use on challenge day.

Direct inoculum was prepared by collecting a 0.5–1 cm^3^ section of the IDS or lesion from each foot. For challenge numbers 4–8, an additional 1 cm^3^ of tissue was collected from each foot with a graded lesion present. Post-tissue collection processing was conducted in an anaerobic chamber (BACTRONEZ Anaerobic Chamber, Cornelius, Oregon, USA). Tissues were minced using sterile forceps and scalpel blades, followed by mixing with MTGE broth (NEOGEN Corporation, Lansing, Michigan, USA) at a ratio of 1:9, tissue to MTGE broth. Tissue suspension was added to soil at a 1:1 ratio and mixed to obtain a homogenous mixture of inoculum. Five mL aliquots of the inoculum were prepared and maintained in an anaerobic environment using an Anaeropack sachet (Mitsubishi, Thermo Scientific, Thermo Fisher Scientific, Waltham, Massachusetts, USA) in an Anaeropack box (Mitsubishi, Thermo Scientific) for up to 3 hours before application to the feet of study elk.

The remaining foot tissue (excluding bones) was processed into 0.5–1.5 cm^3^ pieces to prepare the pen inoculum. For challenge numbers 1–3, a total of 1.4 L of tissue was collected from each case (except for one case in challenge number 2, 0.7 L was collected from the 2 available hooves) for a total of 2.8 L of tissue homogenate. Initially, each case was prepared separately, mixed in a 1:1 ratio with MTGE broth in a clean plastic bag, supplemented with CO_2_ gas for 10 seconds, and closed tightly. Inoculum from each case was then combined and homogenized in a mixer for 1 minute. Next, aliquots for application to each pen were individually prepared by stirring together 0.7 L of the tissue/broth mix and 3.5 L of soil. An aliquot (1.35–1.4 L of tissue/broth mix and 3.5 L of soil) was added to the soil in each pen. Inoculum preparation was similar for challenge numbers 4–8, except tissue collected was increased to 2.1 L for each case (except one case used for challenge number 5 yielded only 1.55 L).

We retrospectively applied a score to describe the quality, i.e., TAHD status, of each case used for treatment inoculum based on the presence of gross lesions, histologic lesions, and detection of treponemes by 16S amplicon sequencing. One point was assigned for the presence of each of 3 attributes and the mean of each case in a challenge was determined. Based on these parameters, the inoculum quality was designated as poor (≤1.5), moderate (2–2.5), or excellent (3) (Table 2).

**Table 2:** Inoculum quality.

Challenge Number	Apparent Graded Lesions				TAHD Diagnosis ^b^	Treponemes ^c^	Challenge Score	Challenge Quality
	By Hoof ^a^							
	H1^d^	H2^e^	H3^f^	H4^g^				
**1 **	0	0	0	IV	-	-	1.5	Poor
	0	0	II	IV	+	-		
**2 **	0	0	0	0	-	-	1.5	Poor
	NA^h^	NA	IV	0	+	+		
**3 **	0	0	0	IV	+	+	3	Excellent
	0	0	IV	I	+	+		
**4 **	0	0	I	III	+	+	3	Excellent
	IV	II	IV	III	+	+		
**5 **	I	0	I	III	+	+	3	Excellent
	0	0	I	II	+	+		
**6 **	0	0	IV	0	+	+	3	Excellent
	0	0	0	III	+	+		
**7 **	III	0	0	0	-	-	1.5	Poor
	0	0	IV	II	-	+		
**8 **	0	0	II	I	+	+	2	Moderate
	II	0	0	0	-	-		

Quality of inoculum prepared from the feet of free-ranging elk with lesions consistent with treponeme-associated hoof disease (TAHD) as assessed for each of 8 challenges of captive study elk. Evaluation of feet from 16 elk (2 elk per challenge) are shown. One point each was assigned for the presence of gross lesions, histologic lesions, and treponemes to obtain an average challenge score. A qualitative ranking of poor (≤1.5), moderate (2–2.5), or excellent (3) was assigned.

^a^ Lesion grades were assigned 0-IV using a previously reported grading system [3].

^b^ Hoof biopsies were diagnosed as TAHD-positive or TAHD-negative using histologic evaluation.

^c^ Presence of treponemes was determined using 16S rRNA gene amplicon sequencing.

^d^ Hoof #1, right front or front hoof #1 if location known.

^e^ Hoof #2, left front or front hoof #2 if location known.

^f^ Hoof #3, right hind or hind hoof #1 if location known.

^g^ Hoof #4, left hind or hind hoof #2 if location known.

^h^ NA = Not available; hoof not present.

### Challenge and sampling of study elk

Elk were experimentally challenged on days post-initial inoculation (dpi) 0, 35, 70, 84, 98, 105, 119, and 133. We immobilized study elk with intramuscular (IM) injection of butorphanol tartrate, azaperone tartrate, and medetomidine HCl (BAM; ZooPharm, Laramie, Wyoming, USA) at about 0.07 mg/kg medetomidine to facilitate handling. Blood was collected for serum mineral analysis and feces were collected for Baermann fecal larval examination and fecal flotation by the Washington Animal Disease Diagnostic Laboratory (WADDL; Pullman, WA, USA). We assessed body condition using a qualitative scale ranging from 1–5 as described previously [17]. The body condition of each elk at each evaluation was assessed at 3 body sites and the mean score was reported.

Each foot (right front, left front, right hind, and left hind or RF, LF, RH, and LH, respectively) was washed with water to remove debris and facilitate visualization. Gross changes and apparent TAHD lesion score (0-IV; [3]) of each foot were recorded. For each individual elk, 2 feet (paired) were randomly selected (RF and LH, or LF and RH) for additional treatment and sample collection. Skin on these feet was scraped with a #21 scalpel blade (Securos Surgical, AmerisourceBergen, Conshohocken, Pennsylvania, USA) to induce an abrasion over a 1 cm^2^ area in the IDS to hasten lesion development as previously described [10] and to provide a sample for 16S amplicon sequencing. Skin scraped from the IDS, and lesion when present, was collected in a sterile cryovial (Axygen, Corning, New York, USA) and stored at -80°C until processed.

**Fig 1:**
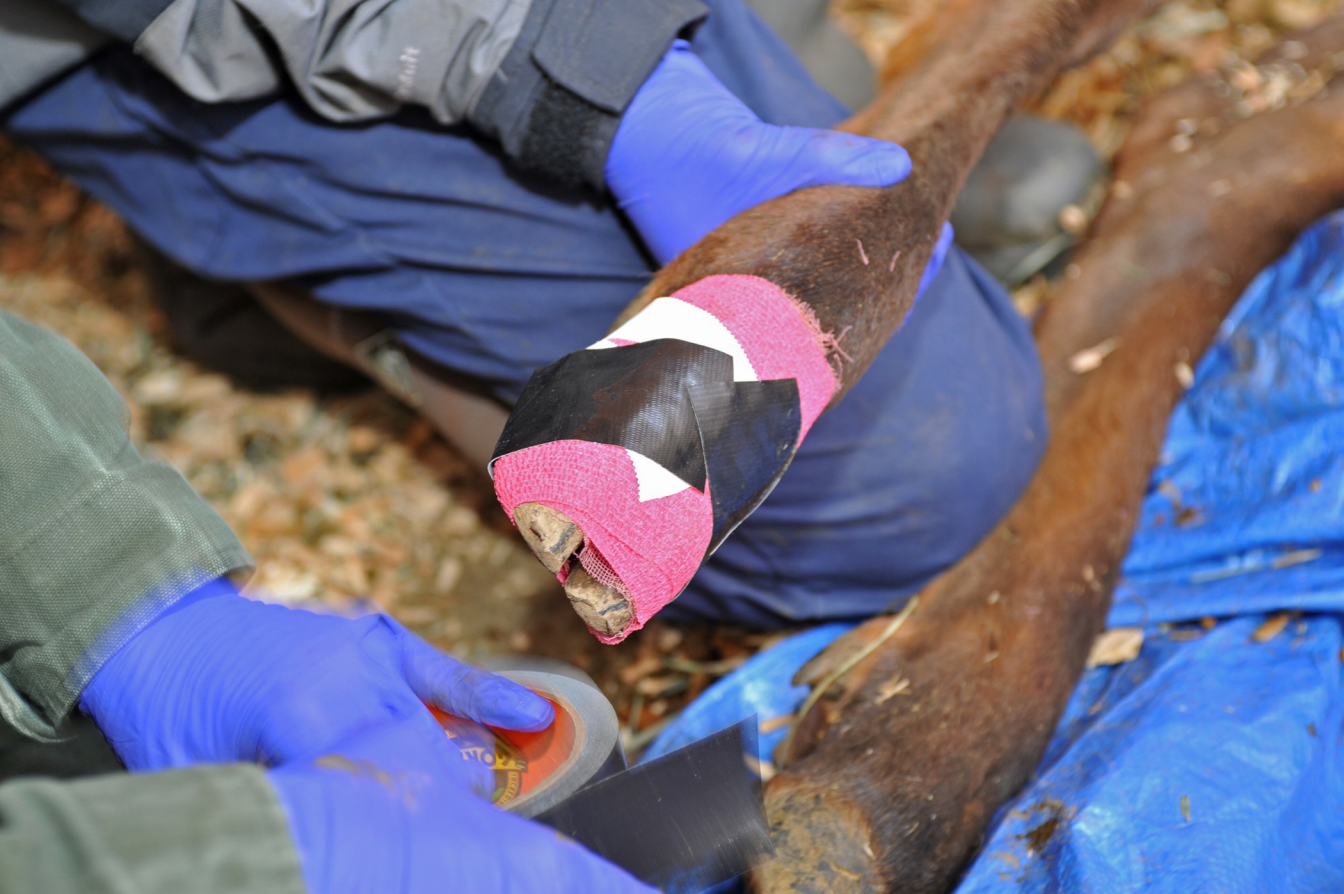
Wraps. Wraps were applied to the feet of captive study elk at 6/8 experimental challenges to extend contact duration of inoculum with the skin of the interdigital space.

Direct inoculum (5 mL) was placed on a sterile gauze saturated in MTGE broth (NEOGEN) and applied to the IDS of each foot. Light wraps were applied to the 2 paired feet of each elk that were treated by scraping (as described above). The wraps, which were intended to temporarily hold inoculum in place and extend contact time with the IDS, were applied on each challenge day except dpi 105 and 133. Feet were wrapped distal to the dew claws with Cling (Jorgenson Laboratories, Inc., Loveland, Colorado, USA) followed by Vetrap (3M Animal Care Products, St. Paul, Minnesota, USA). Gorilla Tape (Gorilla Glue, Cincinnati, Ohio, USA) was placed on the solar surface (Fig 1). A pen inoculum aliquot was also added to the soil in each pen on each of the 8 challenge days. Following collection of samples from elk and application of inoculum, sedation was reversed with IM injection of tolazoline (ZooPharm; about 0.5 mg/kg), naltrexone (ZooPharm; about 0.2 mg/kg), and atipamezole (ZooPharm; about 4 mg/mg medetomidine).

The challenge period concluded when all treatment elk exhibited persistent lesions consistent with TAHD. At the end of the challenge period on dpi 138, sampling and evaluation were conducted as on challenge days. Additionally, we aseptically collected a 5-mm punch biopsy from the IDS adjacent to the skin scraping from the foot with the most gross change or severe lesion on each elk. Prior to biopsy collection, we locally infused the biopsy site with 6 mL of a 50:50 mixture of lidocaine (Hikma Farmacêutica, S.A., Terrugem, Portugal) and bupivacaine (AuroMedics Pharma LLC, East Windsor, New Jersey, USA) and topically applied 75% ethanol with a cotton swab. Meloxicam (1 mg/kg; Zydus Pharmaceuticals USA Inc, Pennington, New Jersey, USA) was administered orally as an analgesic. Following collection, each biopsy was bisected using an aseptic technique. One section was placed in AllProtect tissue reagent (QIAGEN) and stored at -80°C for the extraction of genomic DNA (gDNA) and detection and quantification of treponemes by 16S amplicon sequencing. The second section was placed in 10% neutral buffered formalin for histologic evaluation.

### Monitoring period

Following the challenge period, we conducted a monitoring period to monitor lesion progression. During this period, daily locomotion and behavior assessment continued and elk were immobilized and sampled at monthly intervals as described during the challenge period. An analgesic (Meloxicam, 1 mg/kg) mixed with an apple treat was given orally daily when an animal consistently exhibited a locomotion score of 3.

### Study elk endpoints

Study elk were euthanized to reduce pain and suffering. Euthanasia was indicated when a consistent locomotion score of 3 could not be mitigated at least intermittently (1–2 days/week) after 2 weeks of analgesic administration and consistent behavioral assessment indicated negative impacts (e.g., depressed, reluctant to rise, anorexic, tachypneic) from foot lesions or another debilitating injury or illness. Prior to euthanasia, elk were sedated, blood was collected, and body condition was assessed. Euthanasia was performed using intravenous injection (390 mg pentobarbital sodium and 50 mg of phenytoin sodium/5 kg VetOne Euthanasia Solution, MWI, Boise, Idaho, USA).

### Pathologic examination

Complete diagnostic necropsies of study elk were overseen by a board-certified pathologist at WADDL. Additionally, elk hooves were removed from the carcass and sampled as described for those used for inoculum.

Histologic examinations of biopsies from free-ranging elk hooves used to create inoculum as well as IDS biopsies collected ante- and post-mortem from study elk, and tissues collected post-mortem from study elk were conducted by a pathologist blinded to 16S amplicon sequencing results and treatment group of study elk. Fixed IDS biopsies and post-mortem samples were embedded in paraffin blocks and 3–5 μm sections were cut and stained with H&E for examination. Sections from feet were examined for lesions characteristic of TAHD as previously described [3] and a Warthin-Starry stain was used to confirm the presence of spirochetes within lesions. Tissue Gram stains (Brown and Hopps) were used as needed to help differentiate spirochetes from other argyrophilic bacteria. We defined TAHD cases as those that exhibited suppurative inflammation with argyrophilic spirochetes on histologic examination [3].

### 16S amplicon sequencing

We used 16S amplicon sequencing to detect the presence and proportion of treponemes in the bacterial composition of biopsies and skin scrapings. Genomic DNA was extracted as previously described [2] from IDS biopsies of hooves used to prepare inoculum and from biopsies collected from study elk at the end of the challenge period and at individual elk study endpoints. Skin scrapings that were collected from the IDS of study elk at the time of each challenge were similarly processed for gDNA extraction, except the mincing step used on biopsies was omitted. Briefly, we subjected minced samples or skin scrapings to a proteinase K digestion step prior to processing with the ZymoBIOMICS DNA miniprep kit (Zymo Research, Irvine, California, USA) with minor modifications as described previously. The gDNA samples were submitted to ZymoBIOMICS Targeted Sequencing Service (Zymo Research) for sequencing of the V3-V4 variable region of the 16S rRNA gene using Illumina MiSeq (Illumina Inc., San Diego, California, USA) following the method described previously [2].

### Data analysis

Composition visualization and alpha diversity analyses of the 16S amplicon sequences were performed with QIIME version 1.9.1. Operational taxonomic units (OTUs) were initially identified and assigned with the Zymo Research Database, a 16S rRNA database that is internally designed and curated by ZymoBIOMICS, as a reference database. The objective of 16S amplicon sequencing was to detect and quantify the *Treponema* spp. OTUs in the samples collected from treatment and control elk and from the inoculum used for challenge. The OTUs for which taxonomic assignment was achieved to *Treponema* spp. were reported as such. However, for the OTUs for which the taxonomy assignment was limited to the family *Spirochaetaceae* by ZymoBIOMICS database, we conducted an NCBI-BLAST search to determine their presumptive identity. Using this approach, we refer to OTUs by the most specific taxa assigned by either database. The relative proportions of taxa assigned to genus *Treponema* within the bacterial composition of treatment and control feet and feet used for inoculum were calculated for each sample. In classifying *Treponema* spp., we refer to 3 previously reported BDD phylotypes that have also been associated with TAHD: *Treponema medium/T*. *vincentii*-like, *T*. *phagedenis*-like, and *T*. *pedis* (which includes *T*. *denticola/T*. *putidum*-like OTUs) [4, 7, 18].

Behavior observations for control and treatment groups were compared at 4 time points using the 2-sample test for equality of proportions with continuity correction. Each group was compared to itself at the initial 14 days and final 14 days of the challenge period, as well as the initial 14 days of the monitoring period and final 14 days for each elk prior to endpoints.

We evaluated the impact of lesion progression on body condition and serum mineral levels. Mean body condition score from each elk at each examination (n = 15) up to dpi 280 was analyzed using a 2-way repeated measures analysis of variance (ANOVA) type III test to determine if there was a difference in mean body condition between the experimental groups over the course of the study. To further evaluate a difference in mean body condition between each group at study initiation, end of the challenge period, and at each study elk’s endpoint (n = 6), a Welch’s 2 sample t-test was used. Similarly, to determine if disease had an impact on serum mineral levels over time, a 2-way repeated measures ANOVA type III test and Welch’s 2 sample t-test were used to determine if there was a difference between experimental groups over the course of the study and at study initiation, end of the challenge period, and at each study elk’s endpoint.

Correlation between locomotion scores over time and graded lesions over time from each treatment and control elk was analyzed using linear regression. For each elk, locomotion scores collected 1 day after gross lesion observation on examination days were analyzed using Pearson’s product-moment correlation.

## Results

Elk in both experimental groups (treatment and control) were similar at study initiation. No gross lesions were observed and no disease-associated treponemes were detected in IDS skin scrapings from study elk prior to study initiation (Table 3); however, one rumen-associated treponeme, *T*. *bryantii*, and one treponeme not associated with BDD lesions, *T*. *brennaborense* [19, 20], were each detected at 0.1% of sample bacterial composition in one treatment elk (S1 Table). Behavior (Table 4; p = 1.0) and mean body condition (p = 0.6) did not differ between groups at study initiation. No internal parasites were detected in fecal samples from elk at study initiation. Serum mineral levels did not differ between groups on dpi 0 (S1 Fig; p = 0.6) and were comparable to reference ranges for cattle and deer (*Odocoileus* spp.) except 2 treatment elk were below the reference range for selenium (Se; Table 5) [21]. None of the chemicals analyzed for pesticide or herbicide contamination were detected in soil used in the study.

**Table 3:** Progression of lesions in treatment elk.

Challenge Period													Monitoring Period						
Days Post-initial Inoculation																			
Elk ID		0^a^	7	35^a^	49	70^a^	84^a^	98^a^	105^a^	119^a^	133^a^	138		168	196	224	252	280	308
20–01	Grade	0	0	0	0	0	0^b^	I	0	I^b^	II^b^	II		III	III	III	III		
	Treponemes detected	-		-		-	-	+	+	+	+	+					+^c^		
20–04	Grade	0	0	0	0^b^	0	0	I	II^b^	II	II	II^b^		III	III	III			
	Treponemes detected	-		-		-	+	+	+	+	+	+				+^d^			
20–12	Grade	0	0	0	0	0	0	0	0	I	II	II^b^		II, III^e^					
	Treponemes detected	-		-		-	-	+	+	+	+	+		+^e^					
20–13	Grade	0	0	I	0	0	0	0^b^	0^b^	I	I	II		III	III	III	0	0	0
	Treponemes detected	-		-		-	-	+	-	+	+	+							-

Gross lesions consistent with treponeme-associated hoof disease (TAHD) developed and treponemes were detected in skin scrapings of hooves from captive elk following experimental challenge with inoculum prepared from TAHD-affected hoof material mixed with soil. Results shown are for the foot with the most severe lesion observed on each treatment elk. Gross lesion grades (0-III) are based on a previously reported grading scale [3]. Detection of disease-associated treponemes in skin scrapings, or biopsies on day post-initial inoculation 138 and at the time of euthanasia, using 16S gene amplicon sequencing is indicated by “+” if positive and “-” if negative.

^a^ Inoculum applied to all feet. Foot was wrapped to hold inoculum in place on 2 feet of each elk except on dpi 105 and 133.

^b^ Grade I lesion present on another foot.

^c^ Biopsy collected from grade III lesion at euthanasia on dpi 262.

^d^ Biopsy collected from grade III lesion at euthanasia on dpi 227.

^e^ Grade II lesion on dpi 168. Euthanized on dpi 173 and a grade III lesion was observed and biopsied.

**Table 4:** Behavior change in study elk.

Challenge Period					Monitoring Period			
Initial 14 Days			Final 14 Days		Initial 14 Days		Final 14 Days	
	Control	Treatment	Control	Treatment	Control	Treatment	Control	Treatment
Attitude	1.00	1.00	1.00	0.98	1.00	1.00	1.00	0.93
BAR^a^								
Activity	1.00	0.98	0.96	0.57^1^^,^^2^	0.96	0.84	0.79	0.57^2^
Active								
Respiration	1.00	1.00	1.00	0.93	0.96	0.95	1.00	0.96
Normal^b^								
Appetite	0.93	0.96	1.00	1.00	1.00	0.98	1.00	0.93
Normal^c^								

The proportion of elk by group with normal attitude, activity, respiration, and appetite. The proportion of animal behavior by group was compared for the initial 14 days and final 14 days of the experimental challenge period, as well as the initial 14 days of the monitoring period and final 14 days for each elk prior to endpoints.

^a^ Bright, Alert, Responsive.

^b^ Normal Respiration = 16–24 bpm.

^c^ Normal Appetite = all of daily feed allocation consumed.

^1^ Treatment vs. control at a given time was significantly different (p<0.05).

^2^ Group compared to itself over a period of 2 time points significantly different (p<0.05).

**Table 5:** Mineral levels in study elk.

Group	Elk ID	Time^a^	Se	Cu	Zn	Ca	Fe	Mg	P
Treatment	20–01	DPI 0	0.02	0.85	0.96	97.1	1.3	23	71.3
		DPI 138	0.02	0.86	0.64	89.2	1.3	19	47.8
		Endpoint	0.02	0.9	0.91	90.7	1.2	18	61.2
	20–04	DPI 0	0.05	1	0.93	89.5	1.4	20	54.7
		DPI 138	0.057	0.9	0.69	93.1	1.1	21	63.4
		Endpoint	0.049	0.89	0.63	92.8	1.3	20	63.5
	20–12	DPI 0	0.059	0.66	0.9	96.5	1.3	23	69.2
		DPI 138	0.058	0.77	0.62	92.6	0.96	20	45
		Endpoint	0.048	0.6	0.66	87.2	1.3	20	64.4
	20–13	DPI 0	0.078	0.87	0.91	95.5	1.6	24	56.6
		DPI 138	0.069	0.93	0.52	88.1	1.4	19	40.8
		Endpoint	0.064	0.91	0.79	83.6	1.4	19	52.6
Control	20–03	DPI 0	0.07	0.78	1	90.7	1.5	23	72.8
		DPI 138	0.073	0.79	0.62	93	1.8	21	40.6
		Endpoint	0.064	0.83	1	92.3	1.7	23	68.6
	20–05	DPI 0	0.065	0.86	0.92	89.4	1.5	24	57.2
		DPI 138	0.062	0.83	0.6	87.8	1.3	19	60.8
		Endpoint	0.051	0.73	0.99	91.5	2.2	17	66.3
Reference Ranges			0.053–0.093	0.6–1.2	0.7–1.5	90–128	0.64–1.68	18–32	55–120

Captive study elk serum levels (μg/g) of selenium (Se), copper (Cu), zinc (Zn), calcium (Ca), iron (Fe), magnesium (Mg), and phosphorus (P) at 3 timepoints during the study. Reference ranges are based on values from cattle and deer [21].

^a^ DPI = days post-initial inoculation.

### Challenge period

We defined the challenge period as dpi 0 to 138. The last challenge (challenge number 8) was conducted on dpi 133, when it was observed all treatment elk exhibited persistent gross lesions consistent with TAHD. Other than changes associated with foot lesions, elk remained clinically normal throughout the challenge period. Wraps remained on elk feet from 1 to 14 days, although during this period defects developed on the solar surface of the wrap exposing hooves to pen soil.

Free-ranging elk hooves used to prepare inocula on all challenge days (8/8) exhibited gross lesions suggestive of TAHD (Table 2). Histologic examination confirmed TAHD in at least one of the cases used to prepare inoculum for 7/8 challenges. Similarly, treponemes, including BDD phylotypes and OTUs previously detected in TAHD lesions [2], were detected using 16S amplicon sequencing in at least one of the cases used to prepare inoculum for 7/8 challenges (Table 2; S2 Table). Using our scoring system to evaluate inoculum quality, challenge numbers 3, 4, 5, and 6 were classified as excellent; challenge numbers 1, 2, and 7 as poor; and challenge number 8 as moderate quality (Table 2).

**Fig 2:**
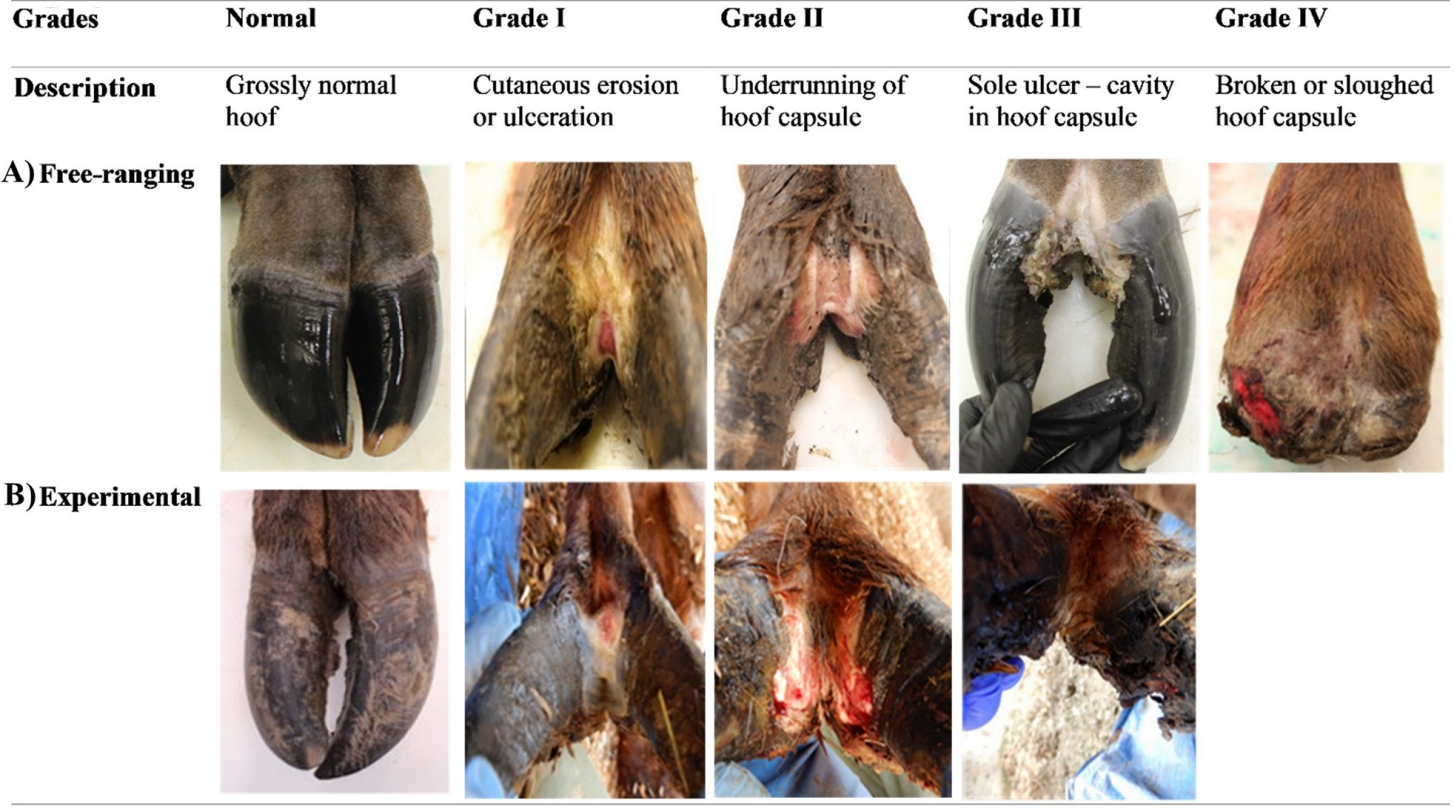
Free-ranging vs. study elk TAHD lesions. Hoof lesions indistinguishable from those in free-ranging elk with treponeme-associated hoof disease (TAHD) developed in study elk following experimental challenge with inoculum prepared from TAHD-affected hoof material mixed with soil. (A) Characteristic TAHD lesions from free-ranging elk graded as I to IV based on a previously described grading system [3] provide a reference for comparison to lesions observed in (B) representative lesions on experimentally infected elk. Lesions progressed through the study period from grade 0 to III.

Following experimental challenges, 4/4 treatment group elk developed gross and histologic lesions characteristic of TAHD (Table 3), while 0/2 control group elk exhibited gross or histologic lesions. Lesion grade was based on gross changes and represent apparent grade without histologic confirmation except when biopsies were collected. The first apparent grade I TAHD lesion was observed, but disease-associated treponemes were not detected, on a wrapped foot at dpi 35 (Table 3). Another grade I lesion was observed in an unwrapped foot at dpi 49. In each of these cases, lesions were transient. Persistent lesions first occurred on dpi 98. By dpi 119, all treatment elk exhibited gross lesions consistent with TAHD. At the end of the challenge period (dpi 138), all treatment elk exhibited grade II lesions on a hind foot (Table 3; Fig 2), with 2/4 also exhibiting a grade I lesion on a front foot. Gross lesions were observed in unwrapped and non-abraded feet, but persistent lesions were observed only in wrapped and abraded feet. No graded lesions were observed on the feet of the control elk throughout the study period.

Other gross abnormalities were observed on the feet of treatment elk prior to the appearance of graded TAHD lesions. Pitting, defined as crateriform (round) shallow depressions in the keratin, was observed in the IDS of both unwrapped and wrapped feet in all treatment elk by dpi 49. Multiple deep coalescing pits formed linear erosions along the medial heel bulb in unwrapped and wrapped feet during dpi 49 to 98. Keratin in the IDS became pale and friable by dpi 98 regardless of the presence of graded lesions. Spontaneous erosions and defects without hyperemia developed at locations in the IDS other than where skin was abraded. However, once developed, these non-hyperemic erosions and defects were subsequently abraded for sample collection during challenges, which may have contributed to lesion development. Grade I lesions, defined as cutaneous erosions and defects with redness/hyperemia (Fig 2), developed in the IDS where spontaneous erosions were previously present and scraped to collect a sample for 16S amplicon sequencing in 8/8 abraded feet. A fetid odor was first noted in the feet of 3/4 treatment elk when grade II lesions were observed. Pitting was observed in both control elk starting at dpi 70 and observed intermittently during the study. Erosions without redness/hyperemia were observed once in a wrapped foot from each control elk, on dpi 84 and 105.

**Fig 3:**
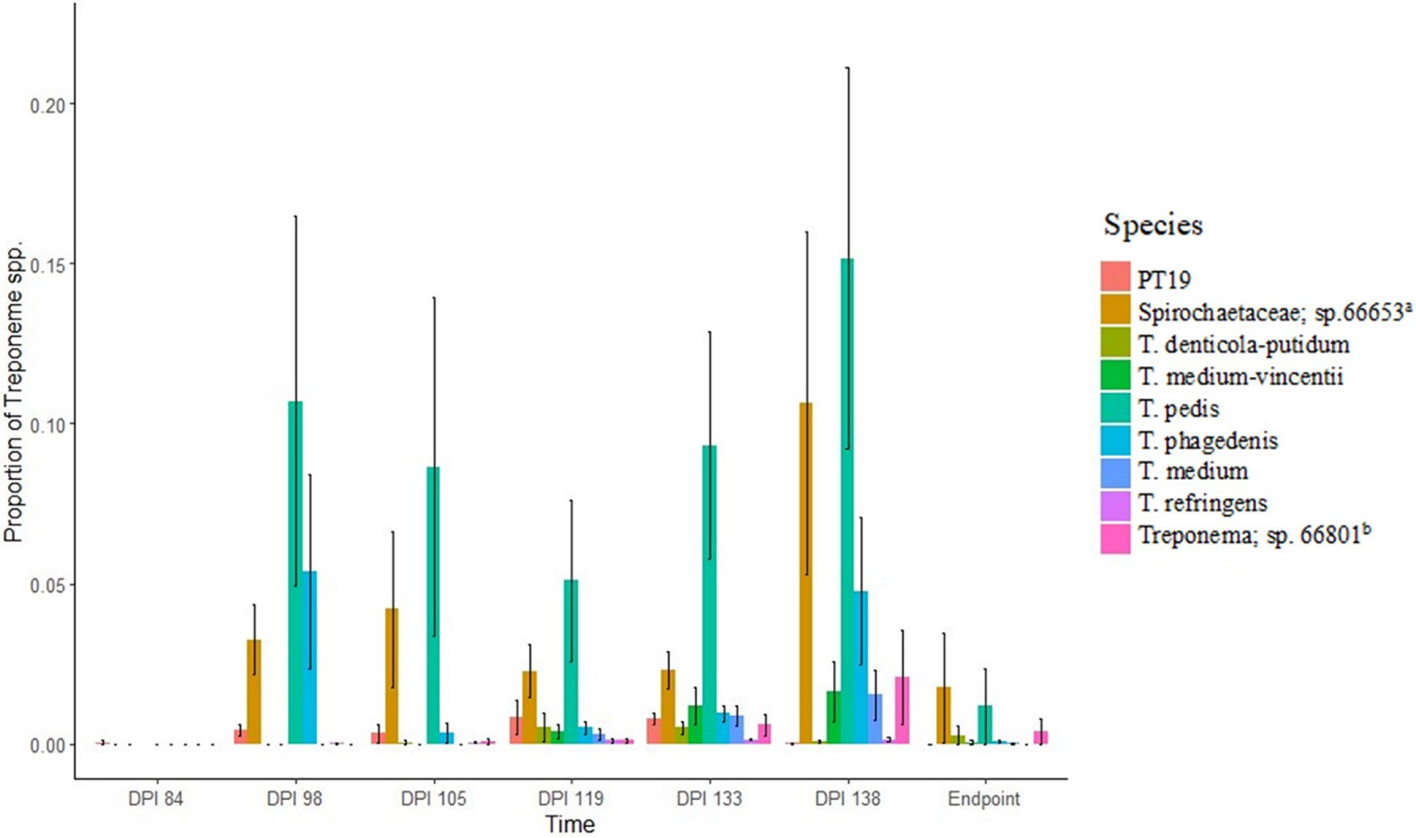
Proportion of treponemes in bacterial composition of foot samples through study period. Mean proportion of *Treponema* species and family *Spirochaetaceae* in the bacterial composition was determined from 16S rRNA gene amplicon sequencing of samples from treatment group elk following experimental challenge with inoculum prepared from TAHD-affected hoof material mixed with soil. Treponemes detected at some point during the study period from every treatment elk are shown. Samples (skin scraping or biopsy) were collected from the interdigital space, and lesion if present, from the foot with the most severe lesion of each elk. Scrapings were collected on sampling days through day post-initial inoculation (dpi) 133, while biopsies were collected on dpi 138 and at individual elk’s endpoints. ^a^ Identified by NCBI BLAST as *T*. *medium*. ^b^ Identified by NCBI BLAST as *T*. *lecithinolyticum*.

We detected treponemes previously associated with TAHD [2] using 16S amplicon sequencing in IDS scrapings from treatment elk feet beginning on dpi 84 (Table 3; Fig 3). Treponemes were detected in at least one foot from each treatment elk by dpi 98. Nine treponeme species were detected from each of the treatment elk (4/4) at a sampling when lesions were persistent (Fig 3). At the end of the challenge period, treponemes were detected in IDS biopsies from all treatment elk, with a mean proportion of 39.48% ± 28.92% of the bacterial composition (Table 3; S1 Table). Disease-associated treponemes were not detected in any sample from control elk throughout the study; however, rumen-associated *T*. *bryantii* [19] and an unclassified *Spirochaetaceae* OTU represented ≤0.2% of the bacterial composition from one elk on dpi 70 (S1 Table). Although treponeme detection was generally coincident with the observation of persistent graded lesions in treatment elk, one OTU previously associated with TAHD [2], *Treponema* sp. clone phylotype 19 (PT19) [22], was detected up to 21 days prior to observation of persistent gross lesions. We detected PT19 and the 3 BDD treponeme phylotypes, as well as *T*. *refringens* which has also been associated with BDD, in persistent lesions from all 4 treatment elk (Fig 3) following detection in inoculum (S1 and S2 Tables). Other treponemes were intermittently detected (S1 Table).

**Fig 4:**
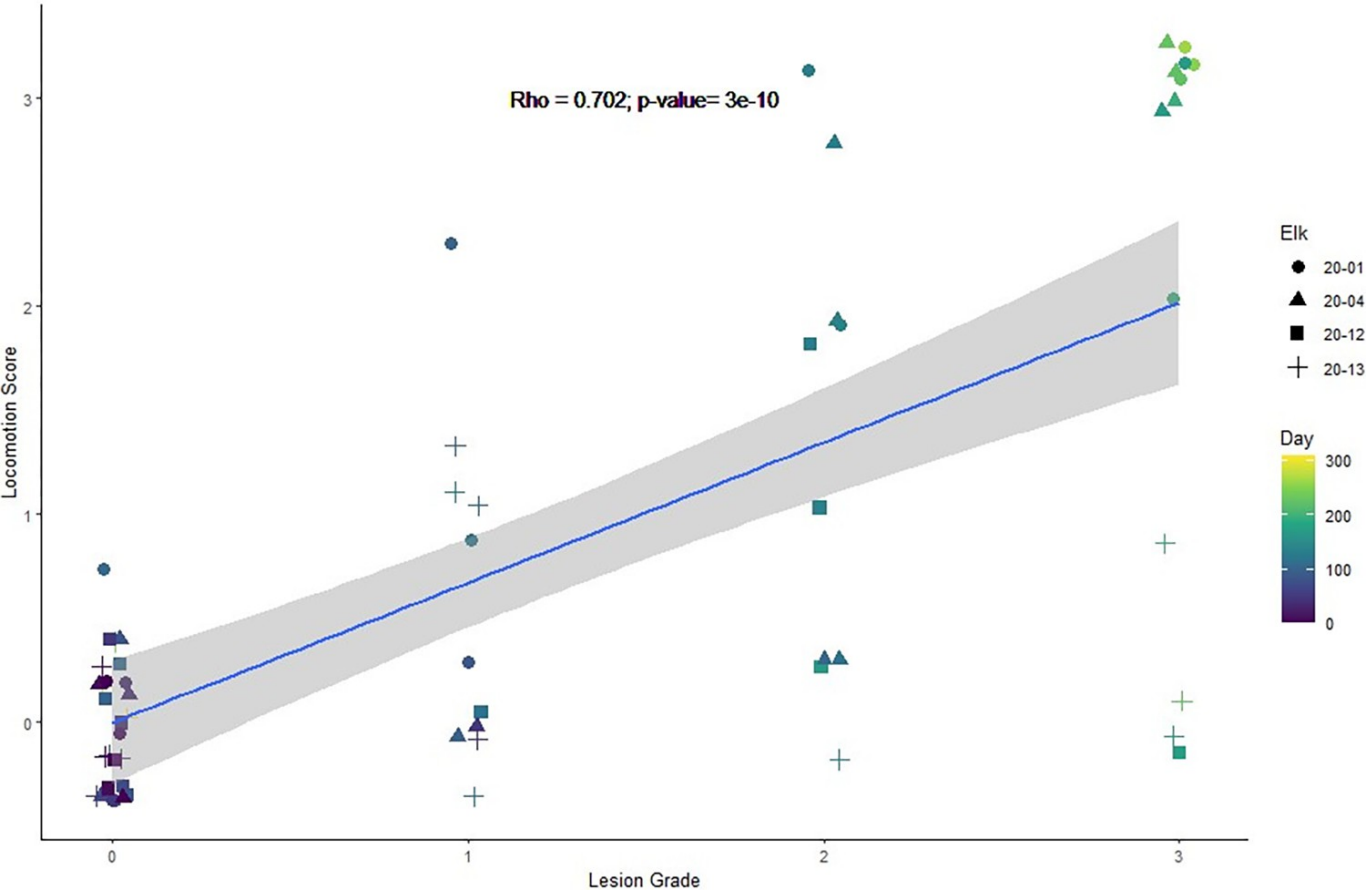
Locomotion score vs. lesion grade. Lameness in treatment elk was correlated with hoof lesion grade (R = 0.702, p≤0.001) following experimental challenge with inoculum prepared from TAHD-affected hoof material mixed with soil. Locomotion scores represent increasing lameness from 0 to 3 with 0 being “sound” locomotion and 3 being severely lame (adapted from [15]). Hoof lesion grades were based on a previously reported grading system [3].

Changes in locomotion were associated with the appearance of foot lesions (Fig 4; S2 Fig). All treatment elk exhibited an imperfect gait (locomotion score of 1) by dpi 100 concurrent with TAHD lesion grade I (Fig 4). By the end of the challenge period (dpi 138), 3/4 treatment elk exhibited lameness (locomotion score of 2). No changes in locomotion were observed in control elk (i.e., locomotion score 0).

**Fig 5:**
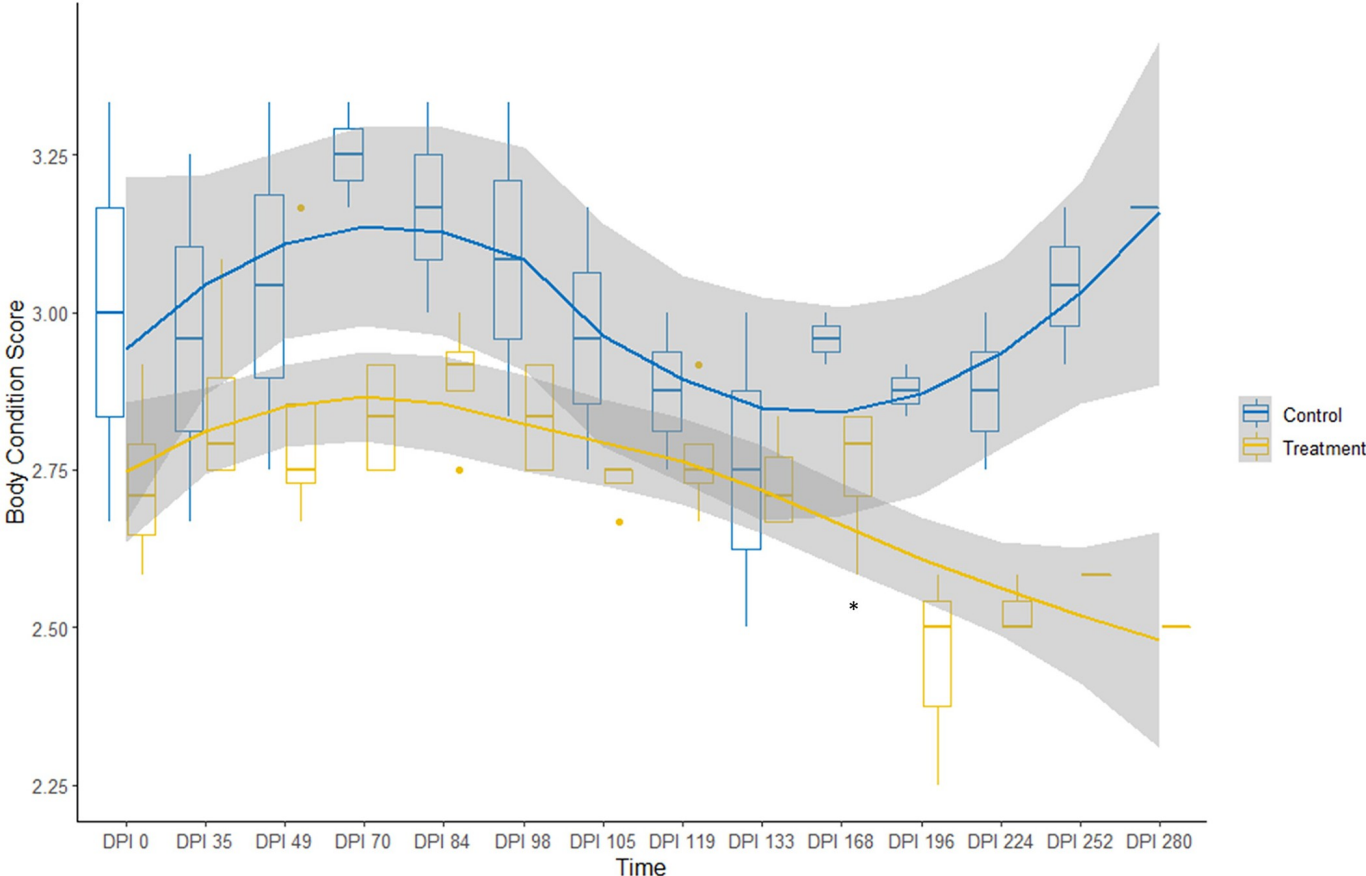
Body condition over time. Body condition of control (blue) and treatment (yellow) groups of elk experimentally exposed to treponeme-associated hoof disease. Body condition of study elk was evaluated on a qualitative scale of 1–5, where 1 represents emaciation and 5 is obese [17]. Experimental groups differed (shown by *) on days post-initial inoculation (dpi) 168, the last timepoint when all study elk were present, and at individual elk’s endpoints.

Despite the development of hoof lesions, elk remained BAR during the challenge period. However, the activity of treatment elk was significantly different from control elk (p≤0.001); treatment elk tended to be bedded more frequently during the last 14 days of the challenge period (p≤0.001) when grade II lesions developed. Respiration remained within normal limits for all elk during the challenge period (Table 4). Elk maintained a normal appetite, consuming all feed provided throughout the challenge period (Table 4). Mean body condition did not differ between the treatment and control group (2.73 and 2.75, respectively) (p = 0.9; Fig 5). Serum mineral levels of Se, copper (Cu), and zinc (Zn) in elk were not significantly different between groups (S1 Fig; Table 5), and no internal parasites were detected at the end of the challenge period.

### Monitoring period

The monitoring period commenced on dpi 138 at which time 4/4 treatment elk exhibited grade II lesions. By dpi 173, lesions had progressed to grade III in all treatment elk (Table 3; Fig 2). Between dpi 173 and 262, 3/4 of treatment elk reached humane endpoints and were euthanized. Two elk were euthanized due to foot lesion impacts on locomotion and decreased activity that could not be mitigated reliably with analgesics, while one elk exhibited clinical signs consistent with pneumonia. Each of these elk had a grade III lesion at the time of death. On the one remaining treatment elk, foot lesions that had been persistent for ≥105 days prior resolved between dpi 224 and dpi 252 and remained at grade 0 until the study conclusion on dpi 308.

All treatment elk reached a locomotion score of 2 by dpi 169 as grade III lesions appeared. Two elk progressed to marked lameness with a locomotion score of 3 by dpi 152, resulting in daily analgesic treatment until endpoints were reached. No changes in locomotion were observed in control elk (i.e., locomotion score 0). Locomotion scores 1 day following examination were strongly correlated with graded lesions observed during examination (R = 0.7, p≤0.001).

Activity level decreased, but changes in attitude, appetite, and respiration were not apparent when treatment elk neared their endpoints. Treatment elk tended to be bedded more often near their endpoints (p = 0.004; Table 4). Elk remained BAR, except when one elk with suspected pneumonia was anorexic and depressed prior to euthanasia. Mean body condition did not differ between groups over the course of the study (p = 0.5); however, mean body condition was lower in treatment than control elk at dpi 168 (p = 0.05), the final timepoint that included all study elk, and at each elk’s endpoint (p = 0.006; Fig 5). Over the course of the study, between groups, Se and Cu levels were not significantly different (p = 0.43 and 0.8, respectively) but Zn was lower in the treatment group (p = 0.05; S1 Fig).

Histology results from biopsies collected at the humane endpoint of study elk exhibited changes consistent with the TAHD case definition in 2/3 elk, while one elk, 20–12, exhibited characteristic inflammation, but spirochetes were not observed in the biopsy (although 16S amplicon sequencing detected treponemes). No biopsy for histologic evaluation was collected from the recovered elk at the conclusion of the study; however, biopsies collected 221 days following the study conclusion (dpi 529) were histologically negative for TAHD. Biopsies collected on dpi 308 from the 2 control elk did not show histologic changes consistent with TAHD. The study was concluded on dpi 308 because no signs of clinical disease, including foot lesions, were observed in the recovered treatment elk over 3 consecutive samplings (56 days) or during the study period in the control elk.

Treponemes were detected using 16S amplicon sequencing from biopsies collected from 3/3 treatment elk sampled at their humane endpoints with grade III lesions (Table 3). At the end of the study period, treponemes detected in IDS biopsies when including all treatment elk, exhibited a mean proportion of 4.30% ± 8.40% of the bacterial composition. All unknown OTU’s identified as *Spirochaetaceae* by ZymoBIOMICS database exhibited ≥90% identity to species of *Treponema*, except sp.66626 which was not identified using BLAST. Treponemes were not detected from the biopsies collected from the 2 control elk at dpi 308 (S1 Table). Similarly, treponemes were not detected in a biopsy from the recovered treatment elk at dpi 529, after the conclusion of the study.

Postmortem examination of the 3 euthanized treatment elk revealed few systemic lesions. One elk (20–12) had severe bronchopneumonia, which was attributed to aspiration during sedation for sampling. One elk (20–04) had enlargement of the right popliteal lymph node, as well as mild chronic multisystemic inflammation, including antler osteomyelitis and thrombosis, which was attributed to low-level septicemia, likely from the hoof lesion on the right hind limb. One elk (20–04) had mild granulomatous pneumonia and eosinophilic bronchiolitis, which was attributed to lungworms, although none were detected. Two elk (20–04 and 20–01) had eosinophilic enteritis, which may have been due to parasites, a hypersensitivity to feed, or an inflammatory bowel disease.

## Discussion

Experimental exposure of elk to soil containing TAHD-positive hooves reproduced gross and histologic lesions indistinguishable from those observed in free-ranging elk with TAHD (Fig 2). Following challenges, lesions in initially normal feet progressed to non-graded changes, e.g., pitting and friability of the IDS, then sequentially from grade I to III. Because elk were euthanized when pain could not be mitigated, we did not observe progression to grade IV lesions; however, gross changes suggested that in time the hoof capsule would have been sloughed. These observations confirm that grade is indeed reflective of lesion progression as hypothesized from single timepoint observations of free-ranging elk [3].

Graded lesions in study elk were observed primarily in the IDS, which is similar to TAHD lesion location in free-ranging elk. Persistent lesions beyond grade I developed only on feet that were abraded and wrapped. Although erosions did not develop at the site of abrasion, persistent grade I lesions developed where erosions were observed and scraped for sample collection. In contrast, in sheep experimentally exposed to TAHD lesion material [13] and cattle exposed to BDD lesion material [10], lesions occurred at the site of abrasion on the ventral pastern and proximal to the heel bulb, respectively. Although in our study non-graded lesions developed without abrasion, because non-graded and graded lesions were scraped for sample collection, it is unclear whether lesions would have progressed similarly without the additional trauma from this scraping. Regardless, free-ranging elk are commonly exposed to environmental conditions that could similarly abrade the IDS, particularly given alterations in skin structure (e.g., friability) we observed prior to development of graded lesions.

Time from initial challenge to persistent grade I lesions in our study elk was similar to that observed in free-ranging elk; however, progression to severe lesions was apparently more rapid. In free-ranging elk, grade I lesions were observed in 3-month-olds, grade II lesions in 7-month-olds, and grade III in 9-month-olds [3]. In our study, timing of lesion appearance and progression was similar among the 4 treatment elk (Table 3). The minimum time from challenge to observation of apparent grade I lesions was 35 days (Table 3) and 2 elk exhibited grade I lesions by dpi 49; however, these lesions resolved by dpi 70. The first lesion that persisted and progressed in severity over time was initially observed on dpi 98. We observed persistent lesions in all treatment elk at dpi 119. Elk were challenged multiple times, limiting our ability to determine whether a particular challenge resulted in lesion development or if multiple challenges were required. Therefore, time to lesion progression represents a maximum estimate. We observed persistent lesions 35–49 days following the first exposures to high-quality inoculum on dpi 70 and dpi 84. Therefore, we speculate that higher-quality challenge inoculum (challenge numbers 3 through 6) may have contributed to the development of persistent lesions.

In the experimental setting, multiple factors likely hastened the rate of lesion progression. Wraps in our study were designed to increase the contact time of the foot with soil-inoculum mixture, increasing the duration of exposure to infectious material. Although our objective in using the wrap was not to create an anaerobic environment as in digital dermatitis experimental challenge studies in cattle [10, 23] and sheep [24], the wrap may have aided in maintaining moisture and reducing oxygen that further promoted lesion development and progression; lesions on wrapped feet in our study progressed to grade III, while lesions on unwrapped feet were transient and none progressed beyond grade I. Because only wrapped feet were abraded, however, our model limits our ability to distinguish the role of wraps from abrasions in the progression of lesion development.

It is unknown whether multiple exposure events are required for lesion development and progression. Experimentally challenged elk were likely exposed to a higher dose when compared to free-ranging elk exposed to soil contaminated by affected elk. In free-ranging elk, the likelihood of exposure is based on factors such as the elk population density, prevalence of TAHD, and conditions that affect pathogen load and survival in soil. We repeatedly challenged study elk to simulate presumed repeated TAHD exposure events in wild elk. We continued with 2 additional challenges after appearance of persistent lesions to optimize conditions to maintain and promote lesion progression. Inoculum preparation was reliant on the opportunistic availability of TAHD positive hooves collected by hunter harvest and management culling. Given that our model used freshly prepared inoculum at each challenge, presence of TAHD compatible gross lesions was the only quality parameter that we could use at the time of challenge. Sampling for histology and 16S amplicon sequencing was conducted on the day of challenge and therefore results were not available. Retrospectively, we found inoculum was categorized as poor or moderate in 4/8 challenges, including poor quality in the initial 2 challenges. The first application of excellent quality inoculum occurred on dpi 70 (Table 2). Quality may have been further impacted by the effects of the freeze-thaw cycle, which likely markedly reduced viable organisms in inoculum [16]. Treponemes are not robust organisms in the environment [11]. Despite these challenges in inoculum preparation, transmission occurred in all exposed elk.

Histologic lesions consistent with TAHD were observed in all treatment elk by the end of the challenge period, but not in control elk. Biopsies collected for histologic evaluation at the end of the challenge period exhibited suppurative inflammation and invasive spirochetes indistinguishable from TAHD in free-ranging elk. Similar results were observed in 2/3 elk that reached humane endpoints. However, in one elk (20–12), inflammation, but not diagnostic spirochetes, was observed histologically despite the presence of a grade III lesion and detection of treponemes using 16S amplicon sequencing. We suspect that the 8-mm punch biopsy was collected from a non-representative location that was likely adjacent to active lesions with spirochetes. Detecting spirochetes histologically can be difficult and, in some cases, they are not observed despite the presence of gross lesions and suppurative inflammation. In TAHD surveillance of free-ranging elk, not all samples in which *Spirochaetaceae* were detected via 16S amplicon sequencing were histologically positive for spirochetes [2]. In BDD lesions, histologic detection of spirochetes is reportedly highest in stage 3 lesions (79%) [25], but >20% of suspect lesions remain unconfirmed.

We focused our 16S amplicon sequencing analysis on treponemes because they are currently considered a hallmark of TAHD [2–4], as well as BDD [7, 25]. The application of 16S amplicon sequencing for pathogen detection provides a comprehensive approach to detection of taxa present in samples along with their relative abundance. Currently, genomic sequences of most treponemes are incomplete and identification is based on partial 16S sequencing. As researchers construct high quality genomes and sequence submissions to publicly available databases grow, the classification of OTUs identified as genus *Treponema* or family *Spirochaetaceae* in this study will likely improve and may lead to discovery and reclassification of novel strains. Similarly, the role of other bacterial families, including but not limited to *Mycoplasmaceae*, *Fusobacteriaceae*, *Porphyromonaceae* that have been reported to be critical components of TAHD along with *Spirochaetaceae* will become clearer [26]. Such investigations were beyond the scope of this study but are warranted as follow-up studies to conclusively demonstrate the etiology of TAHD.

Like gross and histologic lesions, findings from 16S amplicon sequencing in foot scrapings and biopsies from study elk during the challenge period were similar to those in free-ranging elk with TAHD. Treponemes were detected in IDS skin scrapings from all treatment elk when graded lesions were persistently present during the challenge period (Table 3), suggesting that treponemes are integral to lesion persistence. Treponemes were not detected in the first graded lesion we observed on dpi 35, and both lesions we observed on dpi 35 and 49 subsequently disappeared. However, because diagnosis was based solely on gross appearance, these may not have been true grade I lesions.

Treponemes were also detected in biopsies from 3/3 treatment elk with grade III lesions at their endpoints. In the challenge period, treponeme diversity in skin scrapings increased as lesions progressed from 0 to grade II. Despite detection of treponemes in one treatment animal at study initiation and one control animal at dpi 70, these treponemes were not phylotypes associated with BDD and were not detected in tissues used for inoculum (S2 Table), suggesting these species may have been from the environment. Treponemes were detected 2–3 weeks prior and coincident with the observation of persistent lesions. Interestingly, PT19 was detected in early lesions and in 2/4 treatment elk was the only treponeme detected prior to the presence of graded lesions (S1 Table). In a previous study, PT19 was similarly found in feet without graded lesions from free-ranging elk with TAHD diagnosed on other feet [2] leading to speculation that this phylotype may be an important early invader. BDD-associated phylotypes including *T*. *pedis*, *T*. *phagedenis*, *T*. *medium*, and *T*. *refringens* were present in scrapings in persistent lesions in the challenge period, and in biopsies at the end of the challenge period and at endpoints of treatment elk.

Biopsies are the standard tissue collected for metagenomic analysis [2, 25, 27]. We limited the antemortem collection of punch biopsies to 2 per study elk to prevent exacerbating lesion development and out of concern for animal welfare. Biopsies collected from study elk at the end of the challenge period/initiation of the monitoring period and postmortem/end of the study confirmed the presence of treponemes. Skin scrapings collected at other time points during the study provided additional insights on presence of treponemes. These scrapings show promise as a less invasive sampling technique that should be evaluated in the future for concurrence with biopsy findings.

Although 3/4 elk that developed grade III lesions required euthanasia, one elk fully recovered. Recovery is thought to be rare in free-ranging elk and this represents the first published report of recovery from TAHD. Despite the recovery of 1/4 severely affected elk, we caution against reliance on natural recovery as a means of limiting TAHD in free-ranging populations. Our sample of affected elk was small and conditions of captivity (e.g., provision of feed and water, limited locomotion requirements) eased energetic demands and risks of injury or death from predation.

Locomotion affects the survival of free-ranging elk through the need to obtain forage, avoid predators and human disturbance, and for migration. Lameness, as measured by locomotion scores, increased in treatment elk as graded lesions progressed over time (p≤0.001; Fig 4). Similar results in cattle with BDD found a correlation between locomotion and lesion severity [28–30] as well as in CODD, where locomotion worsened with the severity of lesions [31]. Individual variability was observed in the severity of locomotion impairment related to lesion grade, however. Two elk with grade III lesions had locomotion scores of 3 even during analgesic treatment while the 2 other elk with grade III lesions were usually observed with locomotion scores of 2. In the elk that recovered from TAHD, locomotion scores returned to 0 following the resolution of lesions. Locomotion scoring may be useful to managers as a reliable indicator for TAHD-related lameness in free-ranging elk, particularly early in the disease course when gross lesions are not readily apparent from a distance.

Although study elk remained clinically healthy, with the exception of foot lesions and one elk that developed aspiration pneumonia, body condition and activity levels were significantly lower in treatment elk at each elk’s endpoint. Lameness in dairy cattle has also been associated with lower body condition and activity [32]. In our study, a 20% decrease in daily hay fed to each animal at dpi 51 was intended to reproduce seasonal variations in forage available to free-ranging elk during winter [33] and likely contributed to decrease in body condition in both groups. We suspect that loss of body condition detected by the beginning of the monitoring period in treatment elk (Fig 5) was associated with increased energy demands in response to infection. Free-ranging elk experience more substantial impacts from survival stressors than captive elk, so reductions in locomotion and body condition associated with TAHD are likely more detrimental to free-ranging elk.

We anticipated that the concentration of minerals associated with immune response, such as Se, Cu, and Zn, may have been reduced as lesions progressed and inflammation increased [34]; however, no clear evidence of this trend was observed. Serum mineral levels in 2 treatment elk at study initiation were below normal limits of Se established for cattle and deer (Table 5). At the end of the study, Se and Zn were below normal limits in 3 treatment elk and 2 treatment elk, respectively. At the endpoints for each elk, Se and Cu concentrations did not differ between groups but a difference was detected for Zn concentration (Table 5; S1 Fig). Lower hair mineral levels of Se, but not Cu or Zn, were associated with TAHD in free-ranging elk [35]. More research on mineral levels in elk is needed because documentation of serum mineral levels in free-ranging elk is limited [33, 36, 37] and concentrations vary geographically [38]. As a result, reference ranges are based on values in domestic livestock, deer, and captive red deer (*C*. *elaphus*) and elk [21, 33, 36, 37]. Therefore, caution is warranted when comparing to the normal ranges of the mineral content of livestock and captive red deer or elk as it may not be representative of the requirements of free-ranging elk.

On postmortem examination, lesions were primarily limited to the feet of treatment elk. Low-level inflammatory changes sporadically observed in organs may have been associated with bacterial showering from infected feet or alternatively from other bacterial insults. Enlarged lymph nodes were observed in areas draining TAHD-affected feet were not unexpected and indicative of local inflammatory response. No lesions were observed in muscle tissue proximal to the fetlock. Quality and safety of meat from TAHD-affected elk is of concern to hunters that harvest elk. The safety of wild game meat cannot be ensured and varies with individual cases, but in 4 treatment elk with grade III lesions meat quality did not appear to be affected. Importantly, however, poorer meat quality is more likely to be associated with emaciated animals and those with trauma or infection occurring secondary to TAHD, traits that were not present in the study elk euthanized prior to severe debilitation.

Taken together, our results indicate that elk exposed to TAHD-affected hoof material in the soil can develop TAHD and that elk can progress to a debilitated state within 32 weeks following initial pathogen exposure. Lameness and debilitation associated with TAHD are animal welfare concerns. Further, at the population level, the associated risk of mortality may threaten the sustainability of elk populations in areas where TAHD occurs at a high prevalence. As such, management to reduce the negative impacts of TAHD is warranted and urgently needed.

Results from this study can inform wildlife management approaches to control TAHD. Management techniques differ between infectious transmissible diseases and non-communicable diseases, such as exposure to toxins and nutritional diseases. As an environmentally transmissible infectious disease, management approaches will focus on mitigating disease spread by reducing pathogen transmission and identifying and reducing pathogen reservoirs. In dairy environments housing BDD-positive cattle, reservoirs beyond BDD lesions have been challenging to identify [39]. Treponemes are difficult to detect and have been detected in only low abundance in the environment [11, 40]. Improved sampling techniques for treponemes, and potentially other pathogens associated with TAHD, in the soil would help elucidate the role of soil as a reservoir, including the longevity of risk following contamination by TAHD-positive elk.

Although our experimental design sought to replicate indirect transmission, the potential direct animal-to-animal transmission remains unknown and should also be investigated. Further, a more refined model for experimental induction of TAHD would aid in inquiries into host susceptibility, immune response, and the role of one or more treponemes or other bacteria. Although our findings show that captive elk without exposure to other stressors (e.g., nutritional condition, toxin exposure) can develop TAHD following challenges with affected hooves, further research is required to understand the potential contribution of these other stressors in exacerbating disease susceptibility. Based on TAHD surveillance [2] and the knowledge that TAHD is a transmissible infectious disease, a geographic expansion of disease can be inferred, although determining whether cases arise from single or multiple sources due to natural and anthropomorphic factors remains unknown. Future studies can investigate epidemiological questions and model the risk of disease transmission that can be used to refine management to control this emerging disease.

## Supporting information

S1 Fig Serum mineral levels in study elk. Serum mineral levels (μg/g) of captive study elk in control (blue) and treatment (yellow) groups were compared for A) selenium (Se), B) copper (Cu), and C) zinc (Zn) at 3 timepoints during the study: study initiation (dpi 0), monitoring period initiation (dpi 138), and the endpoint of each elk. Reference ranges (red) are based on values from cattle and deer [21, 37]. Statistical differences are indicated with *.
(ZIP)

S2 Fig Locomotion score and graded lesion. Daily locomotion scores were assigned to treatment elk exposed to soil mixed with inoculum from treponeme-associated hoof disease (TAHD) affected feet. Scores represent increasing lameness from 0 to 3 with 0 being “sound” locomotion and 3 being severely lame (adapted from [15]). Locomotion scores increased over the course of the study indicating increased lameness with time and with gross lesion grade. Hoof lesion grades were based on a previously reported grading system [3].
(TIF)

S1 Table Proportion of *Treponema* spp. and *Spirochaetaceae* in study elk. Mean proportion of *Treponema* spp. and *Spirochaetaceae* in the bacterial composition of samples collected from study elk experimentally challenged with inoculum prepared from treponeme-associated hoof disease-affected hoof material (treatment group) or autoclaved hoof material from normal elk (control group) mixed with soil. Samples collected from the interdigital space, and lesion if present, from the foot with the most severe lesion of each elk are shown through time. Scrapings were collected on days post-initial inoculation (dpi) 0, 35, 70, 84, 98, 105, 119, and 133, while biopsies were collected on dpi 138 and designated endpoints. Bacterial composition was determined based on results from 16S rRNA gene amplicon sequencing. ^a^ Detected in other foot.
(DOCX)

S2 Table Proportion of *Treponema* spp. and *Spirochaetaceae* in inoculum. Proportion of treponemes in the bacterial composition of inoculum prepared from the feet of free-ranging elk with lesions consistent with treponeme-associated hoof disease used for each of 8 challenges of captive study elk (2 cases/challenge). Biopsies were collected from the interdigital space, and lesion if present. Bacterial composition was determined based on results from 16S rRNA gene amplicon sequencing.
(DOCX)
