# Supplementary figures and images for: Transmission and lesion progression of treponeme-associated hoof disease in captive elk (Cervus canadensis)
Source: PLoS One. 2023 Aug 10;18(8):e0289764. doi: 10.1371/journal.pone.0289764 (PMC10414667; doi:10.1371/journal.pone.0289764)

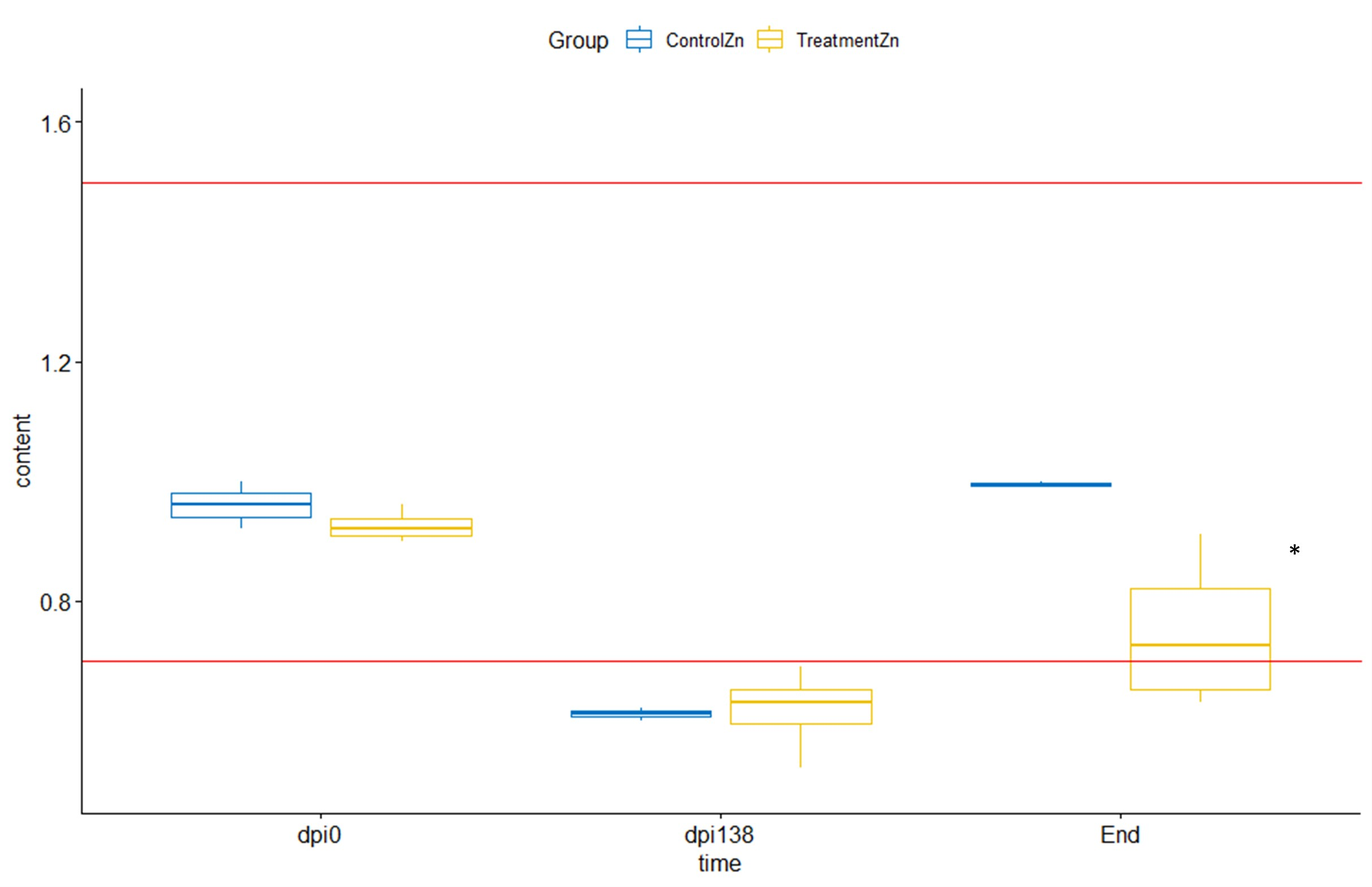

Supplement: S1 Fig — Serum mineral levels (μg/g) of captive study elk in control (blue) and treatment (yellow) groups were compared for A) selenium (Se), B) copper (Cu), and C) zinc (Zn) at 3 timepoints during the study: study initiation (dpi 0), monitoring period initiation (dpi 138), and the endpoint of each elk. Reference ranges (red) are based on values from cattle and deer [21, 37]. Statistical differences are indicated with *. (ZIP) [file pone.0289764.s001.zip › S1C_Fig.tif]

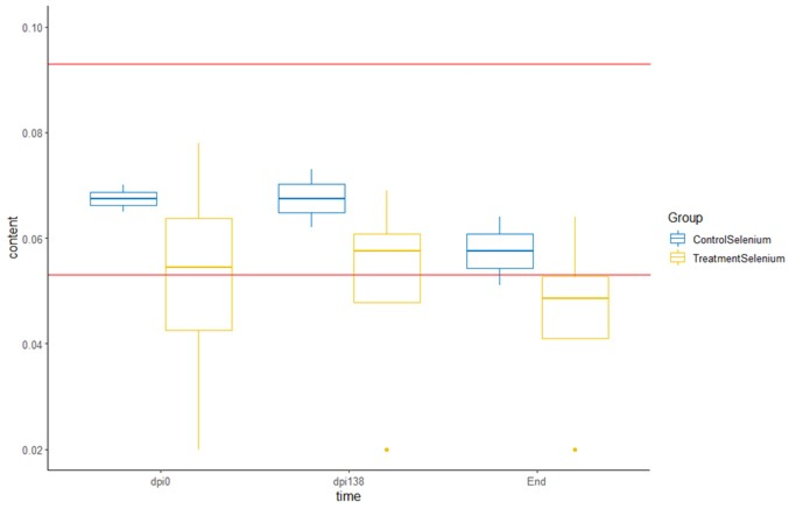

Supplement: S1 Fig — Serum mineral levels (μg/g) of captive study elk in control (blue) and treatment (yellow) groups were compared for A) selenium (Se), B) copper (Cu), and C) zinc (Zn) at 3 timepoints during the study: study initiation (dpi 0), monitoring period initiation (dpi 138), and the endpoint of each elk. Reference ranges (red) are based on values from cattle and deer [21, 37]. Statistical differences are indicated with *. (ZIP) [file pone.0289764.s001.zip › S1A_Fig.tif]

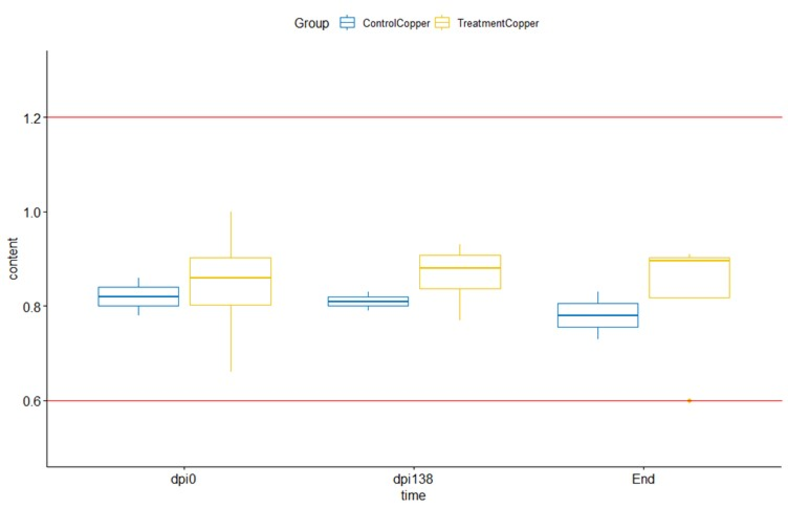

Supplement: S1 Fig — Serum mineral levels (μg/g) of captive study elk in control (blue) and treatment (yellow) groups were compared for A) selenium (Se), B) copper (Cu), and C) zinc (Zn) at 3 timepoints during the study: study initiation (dpi 0), monitoring period initiation (dpi 138), and the endpoint of each elk. Reference ranges (red) are based on values from cattle and deer [21, 37]. Statistical differences are indicated with *. (ZIP) [file pone.0289764.s001.zip › S1B_Fig.tif]

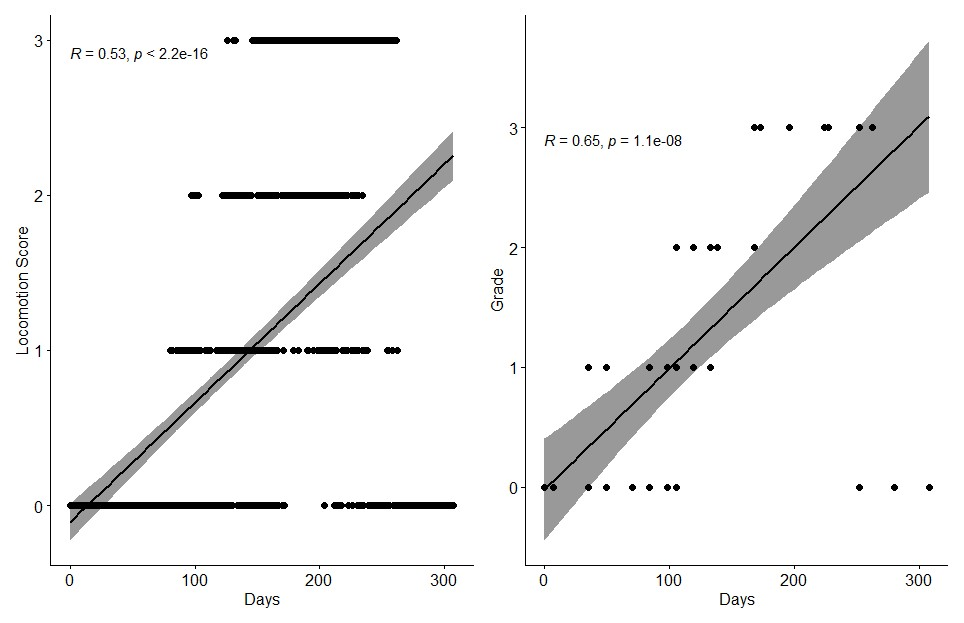

Supplement: S2 Fig — Daily locomotion scores were assigned to treatment elk exposed to soil mixed with inoculum from treponeme-associated hoof disease (TAHD) affected feet. Scores represent increasing lameness from 0 to 3 with 0 being “sound” locomotion and 3 being severely lame (adapted from [15]). Locomotion scores increased over the course of the study indicating increased lameness with time and with gross lesion grade. Hoof lesion grades were based on a previously reported grading system [3]. (TIF) [file pone.0289764.s002.tif]
