# Supplementary material for: Transmission and lesion progression of treponeme-associated hoof disease in captive elk (Cervus canadensis)
Source: PLoS One. 2023 Aug 10;18(8):e0289764. doi: 10.1371/journal.pone.0289764 (PMC10414667; doi:10.1371/journal.pone.0289764)
Supplement: S1 Table — Mean proportion of Treponema spp. and Spirochaetaceae in the bacterial composition of samples collected from study elk experimentally challenged with inoculum prepared from treponeme-associated hoof disease-affected hoof material (treatment group) or autoclaved hoof material from normal elk (control group) mixed with soil. Samples collected from the interdigital space, and lesion if present, from the foot with the most severe lesion of each elk are shown through time. Scrapings were collected on days post-initial inoculation (dpi) 0, 35, 70, 84, 98, 105, 119, and 133, while biopsies were collected on dpi 138 and designated endpoints. Bacterial composition was determined based on results from 16S rRNA gene amplicon sequencing. a Detected in other foot. (DOCX) [file pone.0289764.s003.docx]

S1 Table.

| Elk | Group | Species ID (Zymo) | Species ID (NCBI) | DPI 0 | DPI 35 | DPI 70 | DPI 84 | DPI  98 | DPI 105 | DPI 119 | DPI 133 | DPI 138 | Endpoint |
| --- | --- | --- | --- | --- | --- | --- | --- | --- | --- | --- | --- | --- | --- |
| 20-01 | Treatment | Spirochaetaceae; sp66598 | PT19 | 0.00% | 0.00% | 0.00% | 0.00% | 0.9%^a^ | 1.20% | 0.4%^a^ | 0.3%^a^ | 0.00% | 0.00% |
| 20-04 | Treatment | Spirochaetaceae; sp66598 | PT19 | 0.00% | 0.00% | 0.00% | 0.20% | 0.1%^a^ | 0%^a^ | 0.4%^a^ | 0.8%^a^ | 0.00% | 0.00% |
| 20-12 | Treatment | Spirochaetaceae; sp66598 | PT19 | 0.00% | 0.00% | 0.00% | 0.00% | 0.5%^a^ | 0.10% | 0.10% | 1.00% | 0.10% | 0.00% |
| 20-13 | Treatment | Spirochaetaceae; sp66598 | PT19 | 0.00% | 0.00% | 0.00% | 0.00% | 0.2%^a^ | 0%^a^ | 2.4%^a^ | 1.1%^a^ | 0.00% | 0.00% |
| 20-03 | Control | Spirochaetaceae; sp66598 | PT19 | 0.00% | 0.00% | 0.00% | 0.00% | 0.00% | 0.00% | 0.00% | 0.00% | 0.00% | 0.00% |
| 20-05 | Control | Spirochaetaceae; sp66598 | PT19 | 0.00% | 0.00% | 0.00% | 0.00% | 0.00% | 0.00% | 0.00% | 0.00% | 0.00% | 0.00% |
| 20-01 | Treatment | T. pedis | T. pedis | 0.00% | 0.00% | 0.00% | 0%^a^ | 10.7%^a^ | 0.00% | 12.1%^a^ | 6.4%^a^ | 10.30% | 0.00% |
| 20-04 | Treatment | T. pedis | T. pedis | 0.00% | 0.00% | 0.00% | 0.00% | 26.70% | 13.20% | 2.90% | 15.30% | 20.30% | 4.70% |
| 20-12 | Treatment | T. pedis | T. pedis | 0.00% | 0.00% | 0.00% | 0.00% | 5.40% | 21.40% | 5.10% | 0.70% | 1.30% | 0.00% |
| 20-13 | Treatment | T. pedis | T. pedis | 0.00% | 0.00% | 0.00% | 0%^a^ | 0%^a^ | 0.00% | 0.30% | 14.90% | 28.70% | 0.00% |
| 20-03 | Control | T. pedis | T. pedis | 0.00% | 0.00% | 0.00% | 0.00% | 0.00% | 0.00% | 0.00% | 0.00% | 0.00% | 0.00% |
| 20-05 | Control | T. pedis | T. pedis | 0.00% | 0.00% | 0.00% | 0.00% | 0.00% | 0.00% | 0.00% | 0.00% | 0.00% | 0.00% |
| 20-01 | Treatment | T. phagedenis | T. phagedenis | 0.00% | 0.00% | 0.00% | 0%^a^ | 2.2%^a^ | 0%^a^ | 0.2%^a^ | 1.5%^a^ | 11.40% | 0.20% |
| 20-04 | Treatment | T. phagedenis | T. phagedenis | 0.00% | 0.00% | 0.00% | 0.00% | 13.8%^a^ | 1.3%^a^ | 1.00% | 0.70% | 2.90% | 0.10% |
| 20-12 | Treatment | T. phagedenis | T. phagedenis | 0.00% | 0.00% | 0.00% | 0.00% | 5.5%^a^ | 0.10% | 0.10% | 0.40% | 1.00% | 0.00% |
| 20-13 | Treatment | T. phagedenis | T. phagedenis | 0.00% | 0.00% | 0.00% | 0%^a^ | 0%^a^ | 0.00% | 0.70% | 1.20% | 3.80% | 0.00% |
| 20-03 | Control | T. phagedenis | T. phagedenis | 0.00% | 0.00% | 0.00% | 0.00% | 0.00% | 0.00% | 0.00% | 0.00% | 0.00% | 0.00% |
| 20-05 | Control | T. phagedenis | T. phagedenis | 0.00% | 0.00% | 0.00% | 0.00% | 0.00% | 0.00% | 0.00% | 0.00% | 0.00% | 0.00% |
| 20-01 | Treatment | T.refringens | T.refringens | 0.00% | 0.00% | 0.00% | 0%^a^ | 0.1%^a^ | 0.10% | 0.2%^a^ | 0.1%^a^ | 0.10% | 0.00% |
| 20-04 | Treatment | T.refringens | T.refringens | 0.00% | 0.00% | 0.00% | 0.00% | 0.00% | 0.00% | 0.00% | 0.10% | 0.00% | 0.00% |
| 20-12 | Treatment | T.refringens | T.refringens | 0.00% | 0.00% | 0.00% | 0.00% | 0%^a^ | 0.10% | 0.00% | 0.20% | 0.30% | 0.00% |
| 20-13 | Treatment | T.refringens | T.refringens | 0.00% | 0.00% | 0.00% | 0%^a^ | 0%^a^ | 0.00% | 0.20% | 0.10% | 0.10% | 0.00% |
| 20-03 | Control | T.refringens | T.refringens | 0.00% | 0.00% | 0.00% | 0.00% | 0.00% | 0.00% | 0.00% | 0.00% | 0.00% | 0.00% |
| 20-05 | Control | T.refringens | T.refringens | 0.00% | 0.00% | 0.00% | 0.00% | 0.00% | 0.00% | 0.00% | 0.00% | 0.00% | 0.00% |
| 20-01 | Treatment | Spirochaetaceae; sp66653 | T. medium | 0.00% | 0.00% | 0.00% | 0%^a^ | 4.5%^a^ | 0.00% | 4.5%^a^ | 3.2%^a^ | 13.40% | 0.00% |
| 20-04 | Treatment | Spirochaetaceae; sp66653 | T. medium | 0.00% | 0.00% | 0.00% | 0.00% | 4.60% | 7.70% | 1.00% | 3.00% | 24.50% | 6.90% |
| 20-12 | Treatment | Spirochaetaceae; sp66653 | T. medium | 0.00% | 0.00% | 0.00% | 0.00% | 3.90% | 9.10% | 2.50% | 0.60% | 0.70% | 0.10% |
| 20-13 | Treatment | Spirochaetaceae; sp66653 | T. medium | 0.00% | 0.00% | 0.00% | 0%^a^ | 0%^a^ | 0.00% | 1.10% | 2.40% | 3.90% | 0.00% |
| 20-03 | Control | Spirochaetaceae; sp66653 | T. medium | 0.00% | 0.00% | 0.00% | 0.00% | 0.00% | 0.00% | 0.00% | 0.00% | 0.00% | 0.00% |
| 20-05 | Control | Spirochaetaceae; sp66653 | T. medium | 0.00% | 0.00% | 0.00% | 0.00% | 0.00% | 0.00% | 0.00% | 0.00% | 0.00% | 0.00% |
| 20-01 | Treatment | T.medium | T.medium | 0.00% | 0.00% | 0.00% | 0.00% | 0.00% | 0.00% | 0.80% | 0.60% | 0.20% | 0.00% |
| 20-04 | Treatment | T.medium | T.medium | 0.00% | 0.00% | 0.00% | 0.00% | 0.00% | 0.00% | 0.40% | 1.60% | 3.30% | 0.10% |
| 20-12 | Treatment | T.medium | T.medium | 0.00% | 0.00% | 0.00% | 0.00% | 0.00% | 0.00% | 0.00% | 0.10% | 0.30% | 0.00% |
| 20-13 | Treatment | T.medium | T.medium | 0.00% | 0.00% | 0.00% | 0.00% | 0.00% | 0.00% | 0.00% | 1.20% | 2.30% | 0.00% |
| 20-03 | Control | T.medium | T.medium | 0.00% | 0.00% | 0.00% | 0.00% | 0.00% | 0.00% | 0.00% | 0.00% | 0.00% | 0.00% |
| 20-05 | Control | T.medium | T.medium | 0.00% | 0.00% | 0.00% | 0.00% | 0.00% | 0.00% | 0.00% | 0.00% | 0.00% | 0.00% |
| 20-01 | Treatment | T. denticola-putidum | T. denticola-putidum | 0.00% | 0.00% | 0.00% | 0.00% | 0%^a^ | 0.00% | 1.80% | 0.4%^a^ | 0.00% | 0.00% |
| 20-04 | Treatment | T. denticola-putidum | T. denticola-putidum | 0.00% | 0.00% | 0.00% | 0.00% | 0.00% | 0.20% | 0.30% | 0.90% | 0.10% | 1.10% |
| 20-12 | Treatment | T. denticola-putidum | T. denticola-putidum | 0.00% | 0.00% | 0.00% | 0.00% | 0.00% | 0.00% | 0.00% | 0.00% | 0.10% | 0.00% |
| 20-13 | Treatment | T. denticola-putidum | T. denticola-putidum | 0.00% | 0.00% | 0.00% | 0.00% | 0.00% | 0.00% | 0.00% | 0.70% | 0.10% | 0.00% |
| 20-03 | Control | T. denticola-putidum | T. denticola-putidum | 0.00% | 0.00% | 0.00% | 0.00% | 0.00% | 0.00% | 0.00% | 0.00% | 0.00% | 0.00% |
| 20-05 | Control | T. denticola-putidum | T. denticola-putidum | 0.00% | 0.00% | 0.00% | 0.00% | 0.00% | 0.00% | 0.00% | 0.00% | 0.00% | 0.00% |
| 20-01 | Treatment | T. medium-vincentii | T. medium-vincentii | 0.00% | 0.00% | 0.00% | 0.00% | 0.00% | 0.00% | 0.90% | 0.60% | 0.20% | 0.00% |
| 20-04 | Treatment | T. medium-vincentii | T. medium-vincentii | 0.00% | 0.00% | 0.00% | 0.00% | 0.00% | 0.00% | 0.60% | 2.70% | 3.80% | 0.20% |
| 20-12 | Treatment | T. medium-vincentii | T. medium-vincentii | 0.00% | 0.00% | 0.00% | 0.00% | 0.00% | 0.00% | 0.00% | 0.10% | 0.00% | 0.00% |
| 20-13 | Treatment | T. medium-vincentii | T. medium-vincentii | 0.00% | 0.00% | 0.00% | 0.00% | 0.00% | 0.00% | 0.00% | 1.40% | 2.60% | 0.00% |
| 20-03 | Control | T. medium-vincentii | T. medium-vincentii | 0.00% | 0.00% | 0.00% | 0.00% | 0.00% | 0.00% | 0.00% | 0.00% | 0.00% | 0.00% |
| 20-05 | Control | T. medium-vincentii | T. medium-vincentii | 0.00% | 0.00% | 0.00% | 0.00% | 0.00% | 0.00% | 0.00% | 0.00% | 0.00% | 0.00% |
| 20-01 | Treatment | Treponema sp; 66801 | T. lecithinolyticum | 0.00% | 0.00% | 0.00% | 0.00% | 0.00% | 0.00% | 0.20% | 1.30% | 2.00% | 0.00% |
| 20-04 | Treatment | Treponema sp; 66801 | T. lecithinolyticum | 0.00% | 0.00% | 0.00% | 0.00% | 0.00% | 0.30% | 0.20% | 1.00% | 6.30% | 1.60% |
| 20-12 | Treatment | Treponema sp; 66801 | T. lecithinolyticum | 0.00% | 0.00% | 0.00% | 0.00% | 0.00% | 0.00% | 0.00% | 0.10% | 0.10% | 0.00% |
| 20-13 | Treatment | Treponema sp; 66801 | T. lecithinolyticum | 0.00% | 0.00% | 0.00% | 0.00% | 0%^a^ | 0.00% | 0.00% | 0.00% | 0.00% | 0.00% |
| 20-03 | Control | Treponema sp; 66801 | T. lecithinolyticum | 0.00% | 0.00% | 0.00% | 0.00% | 0.00% | 0.00% | 0.00% | 0.00% | 0.00% | 0.00% |
| 20-05 | Control | Treponemasp; 66801 | T. lecithinolyticum | 0.00% | 0.00% | 0.00% | 0.00% | 0.00% | 0.00% | 0.00% | 0.00% | 0.00% | 0.00% |
| 20-01 | Treatment | Spirochaetaceae; sp66602 | T. pedis | 0.00% | 0.00% | 0.00% | 0.00% | 0.00% | 0.00% | 0.00% | 0.00% | 0.00% | 0.00% |
| 20-04 | Treatment | Spirochaetaceae; sp66602 | T. pedis | 0.00% | 0.00% | 0.00% | 0.00% | 0.00% | 0.00% | 0.20% | 2.80% | 5.60% | 2.20% |
| 20-12 | Treatment | Spirochaetaceae; sp66602 | T. pedis | 0.00% | 0.00% | 0.00% | 0.00% | 0.00% | 0.00% | 0.00% | 0.00% | 0.00% | 0.00% |
| 20-13 | Treatment | Spirochaetaceae; sp66602 | T. pedis | 0.00% | 0.00% | 0.00% | 0.00% | 0.00% | 0.00% | 0.00% | 0.00% | 0.00% | 0.00% |
| 20-03 | Control | Spirochaetaceae; sp66602 | T. pedis | 0.00% | 0.00% | 0.00% | 0.00% | 0.00% | 0.00% | 0.00% | 0.00% | 0.00% | 0.00% |
| 20-05 | Control | Spirochaetaceae; sp66602 | T. pedis | 0.00% | 0.00% | 0.00% | 0.00% | 0.00% | 0.00% | 0.00% | 0.00% | 0.00% | 0.00% |
| 20-01 | Treatment | Spirochaetaceae; sp66702 | T. bryantii | 0.1%^a^ | 0.00% | 0.00% | 0.00% | 0.00% | 0.00% | 0.00% | 0.00% | 0.00% | 0.00% |
| 20-04 | Treatment | Spirochaetaceae; sp66702 | T. bryantii | 0.00% | 0.00% | 0.00% | 0.00% | 0.00% | 0.00% | 0.00% | 0.00% | 0.00% | 0.00% |
| 20-12 | Treatment | Spirochaetaceae; sp66702 | T. bryantii | 0.00% | 0.00% | 0.00% | 0.00% | 0.00% | 0.00% | 0%^a^ | 0.00% | 0.00% | 0.00% |
| 20-13 | Treatment | Spirochaetaceae; sp66702 | T. bryantii | 0.00% | 0.10% | 0.00% | 0.00% | 0.00% | 0.00% | 0.00% | 0.00% | 0.00% | 0.00% |
| 20-03 | Control | Spirochaetaceae; sp66702 | T. bryantii | 0.00% | 0.00% | 0.00% | 0.00% | 0.00% | 0.00% | 0.00% | 0.00% | 0.00% | 0.00% |
| 20-05 | Control | Spirochaetaceae; sp66702 | T. bryantii | 0.00% | 0.00% | 0.20% | 0.00% | 0.00% | 0.00% | 0.00% | 0.00% | 0.00% | 0.00% |
| 20-01 | Treatment | Spirochaetaceae; sp66672 | T. lecithinolyticum | 0.00% | 0.00% | 0.00% | 0.00% | 0.00% | 0.00% | 0.10% | 0.00% | 0.00% | 0.00% |
| 20-04 | Treatment | Spirochaetaceae; sp66672 | T. lecithinolyticum | 0.00% | 0.00% | 0.00% | 0.00% | 0.00% | 0.00% | 0.00% | 0.00% | 0.00% | 0.00% |
| 20-12 | Treatment | Spirochaetaceae; sp66672 | T. lecithinolyticum | 0.00% | 0.00% | 0.00% | 0.00% | 0.00% | 0.00% | 0.00% | 0.00% | 0.00% | 0.00% |
| 20-13 | Treatment | Spirochaetaceae; sp66672 | T. lecithinolyticum | 0.00% | 0.00% | 0.00% | 0.00% | 0.00% | 0.00% | 0.00% | 0.00% | 0.00% | 0.00% |
| 20-03 | Control | Spirochaetaceae; sp66672 | T. lecithinolyticum | 0.00% | 0.00% | 0.00% | 0.00% | 0.00% | 0.00% | 0.00% | 0.00% | 0.00% | 0.00% |
| 20-05 | Control | Spirochaetaceae; sp66672 | T. lecithinolyticum | 0.00% | 0.00% | 0.00% | 0.00% | 0.00% | 0.00% | 0.00% | 0.00% | 0.00% | 0.00% |
| 20-01 | Treatment | Spirochaetaceae; sp66655 | T. brennaborense | 0%^a^ | 0.00% | 0.00% | 0.00% | 0.00% | 0.00% | 0.00% | 0.00% | 0.00% | 0.00% |
| 20-04 | Treatment | Spirochaetaceae; sp66655 | T. brennaborense | 0.00% | 0.00% | 0.00% | 0.00% | 0.00% | 0.00% | 0.00% | 0.00% | 0.00% | 0.00% |
| 20-12 | Treatment | Spirochaetaceae; sp66655 | T. brennaborense | 0.00% | 0.00% | 0.00% | 0.00% | 0.00% | 0.00% | 0.00% | 0.00% | 0.00% | 0.00% |
| 20-13 | Treatment | Spirochaetaceae; sp66655 | T. brennaborense | 0.00% | 0.00% | 0.00% | 0.00% | 0.00% | 0.00% | 0.00% | 0.00% | 0.00% | 0.00% |
| 20-03 | Control | Spirochaetaceae; sp66655 | T. brennaborense | 0.00% | 0.00% | 0.00% | 0.00% | 0.00% | 0.00% | 0.00% | 0.00% | 0.00% | 0.00% |
| 20-05 | Control | Spirochaetaceae; sp66655 | T. brennaborense | 0.00% | 0.00% | 0.00% | 0.00% | 0.00% | 0.00% | 0.00% | 0.00% | 0.00% | 0.00% |
| 20-01 | Treatment | Spirochaetaceae; sp66626 | Unknown | 0.00% | 0.00% | 0.00% | 0.00% | 0.00% | 0.00% | 0.00% | 0.00% | 0.00% | 0.00% |
| 20-04 | Treatment | Spirochaetaceae; sp66626 | Unknown | 0.00% | 0.00% | 0.00% | 0.00% | 0.00% | 0.00% | 0.00% | 0.00% | 0.00% | 0.00% |
| 20-12 | Treatment | Spirochaetaceae; sp66626 | Unknown | 0.00% | 0.00% | 0.00% | 0.00% | 0.00% | 0.00% | 0.00% | 0.00% | 0.00% | 0.00% |
| 20-13 | Treatment | Spirochaetaceae; sp66626 | Unknown | 0.00% | 0.00% | 0.00% | 0.00% | 0.00% | 0.00% | 0.00% | 0.00% | 0.00% | 0.00% |
| 20-03 | Control | Spirochaetaceae; sp66626 | Unknown | 0.00% | 0.00% | 0.00% | 0.00% | 0.00% | 0.00% | 0.00% | 0.00% | 0.00% | 0.00% |
| 20-05 | Control | Spirochaetaceae; sp66626 | Unknown | 0.00% | 0.00% | 0.10% | 0.00% | 0.00% | 0.00% | 0.00% | 0.00% | 0.00% | 0.00% |
| 20-01 | Treatment | Treponema spp; sp66795 | T. pedis | 0.00% | 0.00% | 0.00% | 0.00% | 0.00% | 0.00% | 0.00% | 0.00% | 0.00% | 0.00% |
| 20-04 | Treatment | Treponema spp; sp66795 | T. pedis | 0.00% | 0.00% | 0.00% | 0.00% | 0.00% | 0.00% | 0.20% | 0.30% | 0.40% | 0.00% |
| 20-12 | Treatment | Treponema spp; sp66795 | T. pedis | 0.00% | 0.00% | 0.00% | 0.00% | 0.00% | 0.00% | 0.00% | 0.00% | 0.00% | 0.00% |
| 20-13 | Treatment | Treponema spp; sp66795 | T. pedis | 0.00% | 0.00% | 0.00% | 0.00% | 0.00% | 0.00% | 0.00% | 0.00% | 0.00% | 0.00% |
| 20-03 | Control | Treponema spp; sp66795 | T. pedis | 0.00% | 0.00% | 0.00% | 0.00% | 0.00% | 0.00% | 0.00% | 0.00% | 0.00% | 0.00% |
| 20-05 | Control | Treponema spp; sp66795 | T. pedis | 0.00% | 0.00% | 0.00% | 0.00% | 0.00% | 0.00% | 0.00% | 0.00% | 0.00% | 0.00% |
| 20-01 | Treatment | Treponema spp; sp66799 | T.medium | 0.00% | 0.00% | 0.00% | 0.00% | 0%^a^ | 0.00% | 0.40% | 0.60% | 0.00% | 0.00% |
| 20-04 | Treatment | Treponema spp; sp66799 | T.medium | 0.00% | 0.00% | 0.00% | 0.00% | 0.00% | 0.00% | 0.90% | 1.80% | 0.10% | 0.00% |
| 20-12 | Treatment | Treponema spp; sp66799 | T.medium | 0.00% | 0.00% | 0.00% | 0.00% | 0.00% | 0.00% | 0.00% | 0.00% | 0.00% | 0.00% |
| 20-13 | Treatment | Treponema spp; sp66799 | T.medium | 0.00% | 0.00% | 0.00% | 0.00% | 0.00% | 0.00% | 0.00% | 0.00% | 0.00% | 0.00% |
| 20-03 | Control | Treponema spp; sp66799 | T.medium | 0.00% | 0.00% | 0.00% | 0.00% | 0.00% | 0.00% | 0.00% | 0.00% | 0.00% | 0.00% |
| 20-05 | Control | Treponema spp; sp66799 | T.medium | 0.00% | 0.00% | 0.00% | 0.00% | 0.00% | 0.00% | 0.00% | 0.00% | 0.00% | 0.00% |
| 20-01 | Treatment | Treponema spp; sp66800 | T. denticola | 0.00% | 0.00% | 0.00% | 0.00% | 0.00% | 0.00% | 0.00% | 0.00% | 0.00% | 0.00% |
| 20-04 | Treatment | Treponema spp; sp66800 | T. denticola | 0.00% | 0.00% | 0.00% | 0.00% | 0.20% | 0.70% | 0.10% | 0.20% | 0.40% | 0.00% |
| 20-12 | Treatment | Treponema spp; sp66800 | T. denticola | 0.00% | 0.00% | 0.00% | 0.00% | 0.00% | 0.00% | 0.00% | 0.00% | 0.00% | 0.00% |
| 20-13 | Treatment | Treponema spp; sp66800 | T. denticola | 0.00% | 0.00% | 0.00% | 0.00% | 0.00% | 0.00% | 0.00% | 0.00% | 0.00% | 0.00% |
| 20-03 | Control | Treponema spp; sp66800 | T. denticola | 0.00% | 0.00% | 0.00% | 0.00% | 0.00% | 0.00% | 0.00% | 0.00% | 0.00% | 0.00% |
| 20-05 | Control | Treponema spp; sp66800 | T. denticola | 0.00% | 0.00% | 0.00% | 0.00% | 0.00% | 0.00% | 0.00% | 0.00% | 0.00% | 0.00% |
| 20-01 | Treatment | T. denticola | T. denticola | 0.00% | 0.00% | 0.00% | 0.00% | 0.00% | 0.00% | 0.00% | 0.20% | 0.00% | 0.00% |
| 20-04 | Treatment | T. denticola | T. denticola | 0.00% | 0.00% | 0.00% | 0.00% | 0.00% | 0.00% | 2.90% | 3.00% | 6.90% | 0.00% |
| 20-12 | Treatment | T. denticola | T. denticola | 0.00% | 0.00% | 0.00% | 0.00% | 0.00% | 0.00% | 0.00% | 0.00% | 0.00% | 0.00% |
| 20-13 | Treatment | T. denticola | T. denticola | 0.00% | 0.00% | 0.00% | 0.00% | 0.00% | 0.00% | 0.00% | 0.10% | 0.30% | 0.00% |
| 20-03 | Control | T. denticola | T. denticola | 0.00% | 0.00% | 0.00% | 0%^a^ | 0%^a^ | 0.00% | 0.00% | 0.00% | 0.00% | 0.00% |
| 20-05 | Control | T. denticola | T. denticola | 0.00% | 0.00% | 0.00% | 0.00% | 0.00% | 0.00% | 0.00% | 0.00% | 0.00% | 0.00% |

^a^ = detected in other foot
