# Supplementary material for: Transmission and lesion progression of treponeme-associated hoof disease in captive elk (Cervus canadensis)
Source: PLoS One. 2023 Aug 10;18(8):e0289764. doi: 10.1371/journal.pone.0289764 (PMC10414667; doi:10.1371/journal.pone.0289764)
Supplement: S2 Table — Proportion of treponemes in the bacterial composition of inoculum prepared from the feet of free-ranging elk with lesions consistent with treponeme-associated hoof disease used for each of 8 challenges of captive study elk (2 cases/challenge). Biopsies were collected from the interdigital space, and lesion if present. Bacterial composition was determined based on results from 16S rRNA gene amplicon sequencing. (DOCX) [file pone.0289764.s004.docx]

| Challenge # | Case # | Species ID (Zymo) | Species ID (NCBI) | Proportion | Mean proportion/  challenge |
| --- | --- | --- | --- | --- | --- |
| 1 | 20-90006 | Spirochaetaceae; sp66598 | PT19 | 0.00% | 0.00% |
|  | 20-90007 | Spirochaetaceae; sp66598 | PT19 | 0.00% |  |
| 2 | 2020-16220 | Spirochaetaceae; sp66598 | PT19 | 0.00% | 0.00% |
|  | 2020-16219 | Spirochaetaceae; sp66598 | PT19 | 0.00% |  |
| 3 | 2021-235 | Spirochaetaceae; sp66598 | PT19 | 0.00% | 0.15% |
|  | 2021-236 | Spirochaetaceae; sp66598 | PT19 | 0.30% |  |
| 4 | 2021-571 | Spirochaetaceae; sp66598 | PT19 | 0.00% | 0.00% |
|  | 2021-573 | Spirochaetaceae; sp66598 | PT19 | 0.00% |  |
| 5 | 2021-1678 | Spirochaetaceae; sp66598 | PT19 | 0.60% | 0.30% |
|  | 2021-1666 | Spirochaetaceae; sp66598 | PT19 | 0.00% |  |
| 6 | 2021-1667 | Spirochaetaceae; sp66598 | PT19 | 0.00% | 0.35% |
|  | 2021-1677 | Spirochaetaceae; sp66598 | PT19 | 0.70% |  |
| 7 | 2021-2625 | Spirochaetaceae; sp66598 | PT19 | 0.00% | 0.05% |
|  | 2021-2626 | Spirochaetaceae; sp66598 | PT19 | 0.10% |  |
| 8 | 2021-3282 | Spirochaetaceae; sp66598 | PT19 | 0.00% | 0.00% |
|  | 2021-3283 | Spirochaetaceae; sp66598 | PT19 | 0.00% |  |
| 1 | 20-90006 | T. pedis | T. pedis | 0.00% | 0.00% |
|  | 20-90007 | T. pedis | T. pedis | 0.00% |  |
| 2 | 2020-16220 | T. pedis | T. pedis | 1.80% | 0.90% |
|  | 2020-16219 | T. pedis | T. pedis | 0.00% |  |
| 3 | 2021-235 | T. pedis | T. pedis | 12.20% | 8.60% |
|  | 2021-236 | T. pedis | T. pedis | 5.00% |  |
| 4 | 2021-571 | T. pedis | T. pedis | 15.10% | 8.15% |
|  | 2021-573 | T. pedis | T. pedis | 1.20% |  |
| 5 | 2021-1678 | T. pedis | T. pedis | 0.00% | 11.95% |
|  | 2021-1666 | T. pedis | T. pedis | 23.90% |  |
| 6 | 2021-1667 | T. pedis | T. pedis | 8.10% | 4.25% |
|  | 2021-1677 | T. pedis | T. pedis | 0.40% |  |
| 7 | 2021-2625 | T. pedis | T. pedis | 0.00% | 0.00% |
|  | 2021-2626 | T. pedis | T. pedis | 0.00% |  |
| 8 | 2021-3282 | T. pedis | T. pedis | 0.80% | 0.40% |
|  | 2021-3283 | T. pedis | T. pedis | 0.00% |  |
| 1 | 20-90006 | T. phagedenis | T. phagedenis | 0.00% | 0.00% |
|  | 20-90007 | T. phagedenis | T. phagedenis | 0.00% |  |
| 2 | 2020-16220 | T. phagedenis | T. phagedenis | 0.30% | 0.15% |
|  | 2020-16219 | T. phagedenis | T. phagedenis | 0.00% |  |
| 3 | 2021-235 | T. phagedenis | T. phagedenis | 3.40% | 5.20% |
|  | 2021-236 | T. phagedenis | T. phagedenis | 7.00% |  |
| 4 | 2021-571 | T. phagedenis | T. phagedenis | 0.20% | 0.35% |
|  | 2021-573 | T. phagedenis | T. phagedenis | 0.50% |  |
| 5 | 2021-1678 | T. phagedenis | T. phagedenis | 0.00% | 0.30% |
|  | 2021-1666 | T. phagedenis | T. phagedenis | 0.60% |  |
| 6 | 2021-1667 | T. phagedenis | T. phagedenis | 0.00% | 0.05% |
|  | 2021-1677 | T. phagedenis | T. phagedenis | 0.10% |  |
| 7 | 2021-2625 | T. phagedenis | T. phagedenis | 0.00% | 0.00% |
|  | 2021-2626 | T. phagedenis | T. phagedenis | 0.00% |  |
| 8 | 2021-3282 | T. phagedenis | T. phagedenis | 1.80% | 0.90% |
|  | 2021-3283 | T. phagedenis | T. phagedenis | 0.00% |  |
| 1 | 20-90006 | T. refringens | T. refringens | 0.00% | 0.00% |
|  | 20-90007 | T. refringens | T. refringens | 0.00% |  |
| 2 | 2020-16220 | T. refringens | T. refringens | 0.00% | 0.00% |
|  | 2020-16219 | T. refringens | T. refringens | 0.00% |  |
| 3 | 2021-235 | T. refringens | T. refringens | 0.00% | 0.10% |
|  | 2021-236 | T. refringens | T. refringens | 0.20% |  |
| 4 | 2021-571 | T. refringens | T. refringens | 0.00% | 0.00% |
|  | 2021-573 | T. refringens | T. refringens | 0.00% |  |
| 5 | 2021-1678 | T. refringens | T. refringens | 0.00% | 0.00% |
|  | 2021-1666 | T. refringens | T. refringens | 0.00% |  |
| 6 | 2021-1667 | T. refringens | T. refringens | 0.00% | 0.10% |
|  | 2021-1677 | T. refringens | T. refringens | 0.20% |  |
| 7 | 2021-2625 | T. refringens | T. refringens | 0.00% | 0.00% |
|  | 2021-2626 | T. refringens | T. refringens | 0.00% |  |
| 8 | 2021-3282 | T. refringens | T. refringens | 0.10% | 0.05% |
|  | 2021-3283 | T. refringens | T. refringens | 0.00% |  |
| 1 | 20-90006 | Spirochaetaceae; sp66653 | T. medium | 0.00% | 0.00% |
|  | 20-90007 | Spirochaetaceae; sp66653 | T. medium | 0.00% |  |
| 2 | 2020-16220 | Spirochaetaceae; sp66653 | T. medium | 0.20% | 0.10% |
|  | 2020-16219 | Spirochaetaceae; sp66653 | T. medium | 0.00% |  |
| 3 | 2021-235 | Spirochaetaceae; sp66653 | T. medium | 3.50% | 3.90% |
|  | 2021-236 | Spirochaetaceae; sp66653 | T. medium | 4.30% |  |
| 4 | 2021-571 | Spirochaetaceae; sp66653 | T. medium | 5.60% | 2.80% |
|  | 2021-573 | Spirochaetaceae; sp66653 | T. medium | 0.00% |  |
| 5 | 2021-1678 | Spirochaetaceae; sp66653 | T. medium | 0.00% | 3.20% |
|  | 2021-1666 | Spirochaetaceae; sp66653 | T. medium | 6.40% |  |
| 6 | 2021-1667 | Spirochaetaceae; sp66653 | T. medium | 4.60% | 2.35% |
|  | 2021-1677 | Spirochaetaceae; sp66653 | T. medium | 0.10% |  |
| 7 | 2021-2625 | Spirochaetaceae; sp66653 | T. medium | 0.00% | 0.00% |
|  | 2021-2626 | Spirochaetaceae; sp66653 | T. medium | 0.00% |  |
| 8 | 2021-3282 | Spirochaetaceae; sp66653 | T. medium | 1.30% | 0.65% |
|  | 2021-3283 | Spirochaetaceae; sp66653 | T. medium | 0.00% |  |
| 1 | 20-90006 | T. medium | T. medium | 0.00% | 0.00% |
|  | 20-90007 | T. medium | T. medium | 0.00% |  |
| 2 | 2020-16220 | T. medium | T. medium | 0.30% | 0.15% |
|  | 2020-16219 | T. medium | T. medium | 0.00% |  |
| 3 | 2021-235 | T. medium | T. medium | 0.10% | 0.15% |
|  | 2021-236 | T. medium | T. medium | 0.20% |  |
| 4 | 2021-571 | T. medium | T. medium | 0.00% | 0.00% |
|  | 2021-573 | T. medium | T. medium | 0.00% |  |
| 5 | 2021-1678 | T. medium | T. medium | 0.00% | 0.40% |
|  | 2021-1666 | T. medium | T. medium | 0.80% |  |
| 6 | 2021-1667 | T. medium | T. medium | 0.00% | 0.10% |
|  | 2021-1677 | T. medium | T. medium | 0.20% |  |
| 7 | 2021-2625 | T. medium | T. medium | 0.00% | 0.00% |
|  | 2021-2626 | T. medium | T. medium | 0.00% |  |
| 8 | 2021-3282 | T. medium | T. medium | 0.00% | 0.00% |
|  | 2021-3283 | T. medium | T. medium | 0.00% |  |
| 1 | 20-90006 | T. denticola-putidum | T. denticola-putidum | 0.00% | 0.00% |
|  | 20-90007 | T. denticola-putidum | T. denticola-putidum | 0.00% |  |
| 2 | 2020-16220 | T. denticola-putidum | T. denticola-putidum | 0.20% | 0.10% |
|  | 2020-16219 | T. denticola-putidum | T. denticola-putidum | 0.00% |  |
| 3 | 2021-235 | T. denticola-putidum | T. denticola-putidum | 4.50% | 2.25% |
|  | 2021-236 | T. denticola-putidum | T. denticola-putidum | 0.00% |  |
| 4 | 2021-571 | T. denticola-putidum | T. denticola-putidum | 0.00% | 0.05% |
|  | 2021-573 | T. denticola-putidum | T. denticola-putidum | 0.10% |  |
| 5 | 2021-1678 | T. denticola-putidum | T. denticola-putidum | 0.00% | 0.50% |
|  | 2021-1666 | T. denticola-putidum | T. denticola-putidum | 1.00% |  |
| 6 | 2021-1667 | T. denticola-putidum | T. denticola-putidum | 0.00% | 0.25% |
|  | 2021-1677 | T. denticola-putidum | T. denticola-putidum | 0.50% |  |
| 7 | 2021-2625 | T. denticola-putidum | T. denticola-putidum | 0.00% | 0.00% |
|  | 2021-2626 | T. denticola-putidum | T. denticola-putidum | 0.00% |  |
| 8 | 2021-3282 | T. denticola-putidum | T. denticola-putidum | 0.80% | 0.40% |
|  | 2021-3283 | T. denticola-putidum | T. denticola-putidum | 0.00% |  |
| 1 | 20-90006 | T. medium-vincentii | T. medium-vincentii | 0.00% | 0.00% |
|  | 20-90007 | T. medium-vincentii | T. medium-vincentii | 0.00% |  |
| 2 | 2020-16220 | T. medium-vincentii | T. medium-vincentii | 0.00% | 0.00% |
|  | 2020-16219 | T. medium-vincentii | T. medium-vincentii | 0.00% |  |
| 3 | 2021-235 | T. medium-vincentii | T. medium-vincentii | 0.10% | 0.05% |
|  | 2021-236 | T. medium-vincentii | T. medium-vincentii | 0.00% |  |
| 4 | 2021-571 | T. medium-vincentii | T. medium-vincentii | 0.00% | 0.00% |
|  | 2021-573 | T. medium-vincentii | T. medium-vincentii | 0.00% |  |
| 5 | 2021-1678 | T. medium-vincentii | T. medium-vincentii | 0.00% | 0.00% |
|  | 2021-1666 | T. medium-vincentii | T. medium-vincentii | 0.00% |  |
| 6 | 2021-1667 | T. medium-vincentii | T. medium-vincentii | 0.00% | 0.10% |
|  | 2021-1677 | T. medium-vincentii | T. medium-vincentii | 0.20% |  |
| 7 | 2021-2625 | T. medium-vincentii | T. medium-vincentii | 0.00% | 0.00% |
|  | 2021-2626 | T. medium-vincentii | T. medium-vincentii | 0.00% |  |
| 8 | 2021-3282 | T. medium-vincentii | T. medium-vincentii | 0.00% | 0.00% |
|  | 2021-3283 | T. medium-vincentii | T. medium-vincentii | 0.00% |  |
| 1 | 20-90006 | Treponema sp; 66801 | T. lecithinolyticum | 0.00% | 0.00% |
|  | 20-90007 | Treponema sp; 66801 | T. lecithinolyticum | 0.00% |  |
| 2 | 2020-16220 | Treponema sp; 66801 | T. lecithinolyticum | 0.10% | 0.05% |
|  | 2020-16219 | Treponema sp; 66801 | T. lecithinolyticum | 0.00% |  |
| 3 | 2021-235 | Treponema sp; 66801 | T. lecithinolyticum | 1.20% | 0.65% |
|  | 2021-236 | Treponema sp; 66801 | T. lecithinolyticum | 0.10% |  |
| 4 | 2021-571 | Treponema sp; 66801 | T. lecithinolyticum | 0.00% | 0.05% |
|  | 2021-573 | Treponema sp; 66801 | T. lecithinolyticum | 0.10% |  |
| 5 | 2021-1678 | Treponema sp; 66801 | T. lecithinolyticum | 0.00% | 0.15% |
|  | 2021-1666 | Treponema sp; 66801 | T. lecithinolyticum | 0.30% |  |
| 6 | 2021-1667 | Treponema sp; 66801 | T. lecithinolyticum | 0.00% | 0.00% |
|  | 2021-1677 | Treponema sp; 66801 | T. lecithinolyticum | 0.00% |  |
| 7 | 2021-2625 | Treponema sp; 66801 | T. lecithinolyticum | 0.00% | 0.00% |
|  | 2021-2626 | Treponema sp; 66801 | T. lecithinolyticum | 0.00% |  |
| 8 | 2021-3282 | Treponema sp; 66801 | T. lecithinolyticum | 0.00% | 0.00% |
|  | 2021-3283 | Treponema sp; 66801 | T. lecithinolyticum | 0.00% |  |
| 1 | 20-90006 | Spirochaetaceae; sp66602 | T. pedis | 0.00% | 0.00% |
|  | 20-90007 | Spirochaetaceae; sp66602 | T. pedis | 0.00% |  |
| 2 | 2020-16220 | Spirochaetaceae; sp66602 | T. pedis | 0.10% | 0.05% |
|  | 2020-16219 | Spirochaetaceae; sp66602 | T. pedis | 0.00% |  |
| 3 | 2021-235 | Spirochaetaceae; sp66602 | T. pedis | 2.60% | 1.30% |
|  | 2021-236 | Spirochaetaceae; sp66602 | T. pedis | 0.00% |  |
| 4 | 2021-571 | Spirochaetaceae; sp66602 | T. pedis | 0.00% | 0.00% |
|  | 2021-573 | Spirochaetaceae; sp66602 | T. pedis | 0.00% |  |
| 5 | 2021-1678 | Spirochaetaceae; sp66602 | T. pedis | 0.00% | 0.70% |
|  | 2021-1666 | Spirochaetaceae; sp66602 | T. pedis | 1.40% |  |
| 6 | 2021-1667 | Spirochaetaceae; sp66602 | T. pedis | 0.00% | 0.05% |
|  | 2021-1677 | Spirochaetaceae; sp66602 | T. pedis | 0.10% |  |
| 7 | 2021-2625 | Spirochaetaceae; sp66602 | T. pedis | 0.00% | 0.00% |
|  | 2021-2626 | Spirochaetaceae; sp66602 | T. pedis | 0.00% |  |
| 8 | 2021-3282 | Spirochaetaceae; sp66602 | T. pedis | 0.00% | 0.00% |
|  | 2021-3283 | Spirochaetaceae; sp66602 | T. pedis | 0.00% |  |
| 1 | 20-90006 | Treponema spp; sp66795 | T. pedis | 0.00% | 0.00% |
|  | 20-90007 | Treponema spp; sp66795 | T. pedis | 0.00% |  |
| 2 | 2020-16220 | Treponema spp; sp66795 | T. pedis | 0.00% | 0.00% |
|  | 2020-16219 | Treponema spp; sp66795 | T. pedis | 0.00% |  |
| 3 | 2021-235 | Treponema spp; sp66795 | T. pedis | 0.00% | 0.00% |
|  | 2021-236 | Treponema spp; sp66795 | T. pedis | 0.00% |  |
| 4 | 2021-571 | Treponema spp; sp66795 | T. pedis | 0.00% | 0.00% |
|  | 2021-573 | Treponema spp; sp66795 | T. pedis | 0.00% |  |
| 5 | 2021-1678 | Treponema spp; sp66795 | T. pedis | 0.00% | 0.35% |
|  | 2021-1666 | Treponema spp; sp66795 | T. pedis | 0.70% |  |
| 6 | 2021-1667 | Treponema spp; sp66795 | T. pedis | 0.00% | 0.00% |
|  | 2021-1677 | Treponema spp; sp66795 | T. pedis | 0.00% |  |
| 7 | 2021-2625 | Treponema spp; sp66795 | T. pedis | 0.00% | 0.00% |
|  | 2021-2626 | Treponema spp; sp66795 | T. pedis | 0.00% |  |
| 8 | 2021-3282 | Treponema spp; sp66795 | T. pedis | 0.00% | 0.00% |
|  | 2021-3283 | Treponema spp; sp66795 | T. pedis | 0.00% |  |
| 1 | 20-90006 | Treponema spp; sp66799 | T. medium | 0.00% | 0.00% |
|  | 20-90007 | Treponema spp; sp66799 | T. medium | 0.00% |  |
| 2 | 2020-16220 | Treponema spp; sp66799 | T. medium | 0.20% | 0.10% |
|  | 2020-16219 | Treponema spp; sp66799 | T. medium | 0.00% |  |
| 3 | 2021-235 | Treponema spp; sp66799 | T. medium | 0.80% | 0.65% |
|  | 2021-236 | Treponema spp; sp66799 | T. medium | 0.50% |  |
| 4 | 2021-571 | Treponema spp; sp66799 | T. medium | 0.10% | 0.10% |
|  | 2021-573 | Treponema spp; sp66799 | T. medium | 0.10% |  |
| 5 | 2021-1678 | Treponema spp; sp66799 | T. medium | 0.00% | 0.05% |
|  | 2021-1666 | Treponema spp; sp66799 | T. medium | 0.10% |  |
| 6 | 2021-1667 | Treponema spp; sp66799 | T. medium | 0.00% | 0.05% |
|  | 2021-1677 | Treponema spp; sp66799 | T. medium | 0.10% |  |
| 7 | 2021-2625 | Treponema spp; sp66799 | T. medium | 0.00% | 0.00% |
|  | 2021-2626 | Treponema spp; sp66799 | T. medium | 0.00% |  |
| 8 | 2021-3282 | Treponema spp; sp66799 | T. medium | 0.00% | 0.00% |
|  | 2021-3283 | Treponema spp; sp66799 | T. medium | 0.00% |  |
| 1 | 20-90006 | T. denticola | T. denticola | 0.00% | 0.00% |
|  | 20-90007 | T. denticola | T. denticola | 0.00% |  |
| 2 | 2020-16220 | T. denticola | T. denticola | 0.20% | 0.10% |
|  | 2020-16219 | T. denticola | T. denticola | 0.00% |  |
| 3 | 2021-235 | T. denticola | T. denticola | 0.50% | 0.50% |
|  | 2021-236 | T. denticola | T. denticola | 0.50% |  |
| 4 | 2021-571 | T. denticola | T. denticola | 0.00% | 0.00% |
|  | 2021-573 | T. denticola | T. denticola | 0.00% |  |
| 5 | 2021-1678 | T. denticola | T. denticola | 0.00% | 0.30% |
|  | 2021-1666 | T. denticola | T. denticola | 0.60% |  |
| 6 | 2021-1667 | T. denticola | T. denticola | 0.00% | 0.05% |
|  | 2021-1677 | T. denticola | T. denticola | 0.10% |  |
| 7 | 2021-2625 | T. denticola | T. denticola | 0.00% | 0.00% |
|  | 2021-2626 | T. denticola | T. denticola | 0.00% |  |
| 8 | 2021-3282 | T. denticola | T. denticola | 0.00% | 0.00% |
|  | 2021-3283 | T. denticola | T. denticola | 0.00% |  |
